# Increased Carbon Partitioning to Secondary Metabolites Under Phosphorus Deficiency in *Glycyrrhiza uralensis* Fisch. Is Modulated by Plant Growth Stage and Arbuscular Mycorrhizal Symbiosis

**DOI:** 10.3389/fpls.2022.876192

**Published:** 2022-06-02

**Authors:** Wei Xie, Angela Hodge, Zhipeng Hao, Wei Fu, Lanping Guo, Xin Zhang, Baodong Chen

**Affiliations:** ^1^State Key Laboratory of Urban and Regional Ecology, Research Center for Eco-Environmental Sciences, Chinese Academy of Sciences, Beijing, China; ^2^College of Resources and Environment, University of Chinese Academy of Sciences, Beijing, China; ^3^Department of Biology, University of York, York, United Kingdom; ^4^State Key Laboratory Breeding Base of Dao-di Herbs, National Resource Center for Chinese Materia Medica, China Academy of Chinese Medical Sciences, Beijing, China

**Keywords:** phosphorus deficiency, mycorrhizal symbiosis, secondary metabolites, non-structural carbohydrates, plant growth stage, stress response

## Abstract

Phosphorus (P) is one of the macronutrients limiting plant growth. Plants regulate carbon (C) allocation and partitioning to cope with P deficiency, while such strategy could potentially be influenced by plant growth stage and arbuscular mycorrhizal (AM) symbiosis. In a greenhouse pot experiment using licorice (*Glycyrrhiza uralensis*) as the host plant, we investigated C allocation belowground and partitioning in roots of P-limited plants in comparison with P-sufficient plants under different mycorrhization status in two plant growth stages. The experimental results indicated that increased C allocation belowground by P limitation was observed only in non-AM plants in the early growth stage. Although root C partitioning to secondary metabolites (SMs) in the non-AM plants was increased by P limitation as expected, trade-off patterns were different between the two growth stages, with C partitioning to SMs at the expense of non-structural carbohydrates (NSCs) in the early growth stage but at the expense of root growth in the late growth stage. These changes, however, largely disappeared because of AM symbiosis, where more root C was partitioned to root growth and AM fungus without any changes in C allocation belowground and partitioning to SMs under P limitations. The results highlighted that besides assisting with plant P acquisition, AM symbiosis may alter plant C allocation and partitioning to improve plant tolerance to P deficiency.

## Introduction

In plants, carbon (C) allocation to organs and partitioning among components in the organ plays fundamental roles in plant adaptation to environmental stresses (Chapin, 1991). Phosphorus (P) is one of the main nutrients limiting plant growth because of its low mobility in soil (López-Arredondo et al., 2014). Plants have evolved various strategies to maximize P acquisition efficiency (PAE) and P utilization efficiency (PUE) and cope with P deficiency (López-Arredondo et al., 2014). These strategies include morphological responses (e.g., root architecture modification) (Hodge et al., 2009; Parra-Londono et al., 2018), physiological adaptation (e.g., organic anion and acid phosphatase secretion) (Wang and Lambers, 2020), molecular responses such as induction of *P transporter* (*PT*) gene expression (Ham et al., 2018), and changes in root–microbe interactions such as root colonization with mycorrhizal fungi (Smith et al., 2003, 2011) to increase P acquisition from soil. For example, plants would allocate more C belowground to support root growth and mycorrhizal symbiosis development when the availability of soil resources such as P is low (Glynn et al., 2007; Konvalinková et al., 2017). Besides, plants can remobilize P from different P-containing substances to optimize P utilization (López-Arredondo et al., 2014). Previous studies demonstrated that plants increased tissue C partitioning from non-structural carbohydrates (NSCs; e.g., soluble sugar and starch) toward secondary metabolites (SMs), such as flavonoids, under P limitation (Sampedro et al., 2011; Liu et al., 2016; Shinde et al., 2018; Mo et al., 2019). Rather than consuming P, secondary metabolism can recycle P from phosphate esters and produce reducing equivalents to scavenge free radicals that are induced by P deficiency (Malhotra et al., 2018; Nasr Esfahani et al., 2021). However, according to the growth-differentiation balance hypothesis (GDBH) (Herms and Mattson, 1992), trade-offs of C allocation and partitioning between growth and secondary metabolism exist. Therefore, increased C allocation to roots and partitioning to SMs may result in less C translocation to NSCs that are required for energy storage and plant growth or yield for crop plants (Huang et al., 2017, 2019).

Arbuscular mycorrhizal (AM) fungi, as one of the key symbiotic microbes in soil, can form symbiotic associations with around two-thirds of terrestrial plant species (Hodge et al., 2010; Kiers et al., 2011). On one hand, AM fungi can efficiently improve plant P uptake from soil under P limitation, which is one of the best characterized benefits of AM symbiosis to host plant (Smith and Read, 2008; Albornoz et al., 2021). However, this comes at a cost, with plants having to allocate up to 20% of assimilated C to support their fungal symbionts (Parniske, 2008). On the other hand, the improved plant P uptake by AM symbiosis depends on mycorrhizal status. That is, for host plants, the cost (in terms of C delivered to AM fungi) and benefit (in terms of P acquisition) change over the progress of mycorrhization, and the activity and function of AM fungi are highly relevant to mycorrhizal structure (such as arbuscules) development (Schweiger et al., 2014; Ven et al., 2020). Limited P absorption ability but high C allocation to AM fungi has been demonstrated in the early mycorrhization stage, resulting in depressed plant growth (Miller et al., 2014). Moreover, soil nutrient availability and plant nutrient demand varied with plant growth stage, resulting in varied plant responses to P deficiency (Wang et al., 2018; Zhao et al., 2021). Therefore, despite increased P uptake from soil, mycorrhizal plants may still need internal P recycling to improve PUE by regulating C allocation and partitioning among C pools to cope with P deficiency, particularly for young and rapidly growing plants. Previous studies demonstrated that mycorrhizal plants enhance C allocation from shoots to roots (Boldt et al., 2011; Řezáčová et al., 2018; Andrino et al., 2021) and alter the concentration and composition of primary and secondary metabolites, particularly under P limitation (Gerlach et al., 2015; Schweiger and Müller, 2015; Adolfsson et al., 2017), suggesting that internal P reutilization by regulating C balance between NSCs and SMs plays important roles in AM plant adaption to P limitation.

Although the GDBH model has been well-established to interpret the relationship between plant growth and secondary metabolism under nutrient limitation (Herms and Mattson, 1992) and trade-offs in C allocation to functional C pools, such as growth, NSCs and SMs, under environmental stresses have been identified (Huang et al., 2017, 2019), how plant growth stage and AM symbiosis interactively affect plant C allocation and partitioning to enhance plant tolerance to P limitation is largely unknown. Answering this question is important for sustainable crop cultivation, as C allocation not only affects the development and functionality of AM symbiosis (Smith and Read, 2008) but also influences crop yield and plant resistance against future stresses (Kleczewski et al., 2010; Huang et al., 2017, 2019).

Licorice (*Glycyrrhiza uralensis* Fisch.) is a perennial leguminous medicinal plant species owing to its importance in extraction of pharmacodynamic metabolites including flavonoids (e.g., liquiritin) and saponins (e.g., glycyrrhizin), both of which are C-based SMs and the key components in licorice roots (Kitagawa, 2002; Hayashi and Sudo, 2009). The market demand of licorice has been increasing rapidly in recent years because of gradual extinction of wild licorice plants and scarcity of high-quality cultivated licorice. For example, the annual transaction value of licorice extract had grown from United States $62.9 million in 1997 to 157.1 million in 2017 (Cheng et al., 2020). In addition to the pharmacodynamic value, licorice plants are important food industrial raw materials because of the high sweetness of glycyrrhizin in their roots. Although licorice widely grows in arid and semi-arid regions globally and shows high tolerance to drought, salt, and alkali stress because of its evolved root system (Zhou and Jin, 2016), it has a high P demand, especially in the early growth stage. Thus, licorice growth is often limited by low soil P availability (Hayashi and Sudo, 2009; Zhou and Jin, 2016; Xie et al., 2019). Root SMs, especially flavonoids, may play important roles in helping licorice plants to cope with P deficiency (Pourcel et al., 2007). Previous studies demonstrated that licorice roots could be intensively colonized by AM fungi in soil (Dang et al., 2021), and that AM inoculation not only improved licorice growth but also facilitated SM (e.g., flavonoids and saponins) accumulation under P limitation (Chen et al., 2017; Xie et al., 2018). In this study, licorice was used as the model plant, and responses of non-mycorrhizal plants and plants colonized with AM fungus *Rhizophagus irregularis* to P deficiency were explored from the perspective of C allocation at the whole plant level and partitioning at the root component level. We focused on the trade-offs of C partitioning in roots, as roots are associated with nutrient uptake, plant C translocation to AM symbiont, and active compound accumulation, thus playing critical roles in licorice growth and development under nutrient stress. Two harvests were conducted based on the phenological state of annual licorice growth, with the early growth stage at approximately 60 days and the late growth stage at 120 days after seedling emergence (Zhou and Jin, 2016).

This study hypothesized that (1) increased C allocation belowground and root C partitioning to SMs by P limitation could become more evident in elder plants versus young plants; (2) mycorrhizal plants could show similar response with non-mycorrhizal plants in the early growth stage, with more C allocation belowground and root C partitioning to SMs under P limitation, but these responses could be less significant or even disappear in the late growth stage; and (3) root C partitioning among SMs, NSCs, and root growth could exhibit different patterns between mycorrhizal and non-mycorrhizal plants and between plant growth stages. Tissue C, N, and P concentrations and biomass were measured to evaluate plant performance. Changes in the proportion of NSCs (including sucrose, soluble sugars, and starch) and SMs (including glycyrrhizin, liquiritin, total flavonoids, and total saponins) of plant shoots and roots, and the expression of sucrose transfer family genes (*SUT*) were used to evaluate plant C allocation and partitioning. Furthermore, to gain insights into the effects of P limitation and plant growth stage on AM function, mycorrhizal colonization, the relative abundance of *R. irregularis* in roots (represented as *RiTEF* expression), and the expression of symbiosis-related genes such as *RiMST2* and *RiPT1*, which participate in the exchange of C for P between symbiotic partners (Helber et al., 2011), were also assessed.

## Materials and Methods

### Growth Substrate

Soil with low fertility was collected from the top layer (*ca.* 0–20 cm) of an uncultivated land in Erdos (39°89′N, 110°1′E), Inner Mongolia, China. The soil had a pH of 7.69 (1:2.5 soil to water), an organic matter content of 14.44 g kg^–1^, and an extractable P (with 0.5 M NaHCO_3_, pH 8.5) content of 6.54 mg kg^–1^. A mixture (2:1 w/w) of soil and quartz sand (<2 mm) was used as the growth substrate. Before mixing, the soil was passed through a 2-mm sieve and sterilized by γ-radiation (20 kGy). The quartz sand was autoclaved at 121°C for 1 h for two consecutive days to inactivate mycorrhizal propagules. Before the experiment, the mixture was supplemented with basal nutrients including N and K as follows: 120 mg kg^–1^ NH_4_NO_3_-N, and 120 mg kg^–1^ (KH_2_PO_4_ + K_2_SO_4_)-K as aqueous solution. For convenience, this mixture is hereafter referred to as “soil.”

### Biological Materials

The inoculum of AM fungus *R. irregularis* Schenck & Smith BGC AH01, consisting of colonized roots of *Sorghum bicolor*, spores (*ca*. 60 spores g^–1^ soil), and hyphae in a sandy soil medium, was provided by the Beijing Academy of Agriculture and Forestry, China. Seeds of licorice (*G. uralensis* Fisch.) were collected from a licorice cultivation base in Minqin County, Gansu province, China. The seeds were manually screened to ensure quality (i.e., full and free of any insect incision), immersed in H_2_SO_4_ (50%) for 30 min, surface-sterilized with 10% H_2_O_2_, and washed several times with Milli-Q water before pre-germination on moist filter paper in the dark (at 25°C for 2 days. Germinated seeds with a uniform radicle of 1 cm were used in the experiment.

### Experimental Design and Plant Growth Conditions

The experiment was conducted in a randomized block design with four treatments: mycorrhizal plants (+M) or non-mycorrhizal plants (−M) were grown at each P addition level, namely, low P (LP) with only basal P application (30 mg kg^–1^) and high P (HP) with 170 mg kg^–1^ P application. Based on the previous study, plants were under P limitation and sufficiency at these two P addition levels (Xie et al., 2019). Phosphorus was added as an aqueous solution of KH_2_PO_4_ into the soil and mixed homogeneously before sowing. Because our aim was to compare the performances and differences of -M and +M plants in terms of C allocation and partitioning pattern under LP versus HP conditions, in this context, there were two controls in this study, i.e., non-mycorrhizal (-M) and P sufficiency conditions (HP).

In the +M treatments, 40 g of the AM inoculum was carefully mixed with 400 g of soil before filling plastic pots (diameter, 15 cm; height 14 cm) that were previously filled with 800 g of sterilized soil. The −M treatments received an equivalent amount of inoculum sterilized at 121°C for 30 min, together with 10 ml of an inoculum filtrate (passed through a 20-μm filter to remove AM fungal propagules) for starting microbial communities (except for AM fungi) to be comparable (Hodge, 2001).

Three pre-germinated seeds were sown in each pot and cultivated in a growth chamber at 25°C/20°C (light/dark) and 16-h/8-h (light/dark) intervals. Two weeks later, the seedlings were thinned so that two size-matched seedlings remained in each pot. After 10 days, the pots were transferred to a greenhouse at Beijing Forestry University. The greenhouse had an average day and night temperatures of 25–30 and 20–22°C, respectively, with 60–80% relative humidity during the plant growth period (May 2018–September 2018). The plants were watered daily with deionized water, and the pots were maintained with 16% soil moisture on a dry weight basis (*ca*. 75% of field water capacity) by regular weighing.

### Plant Harvest and Mycorrhizal Colonization

Plants were destructively harvested 60 (early growth stage) and 120 (late growth stage) days after inoculation (DAI), with four replicates per treatment at each harvest. At each harvest, two plants per pot were summed for analysis as one biological replicate. Plant tissues were separated into aboveground (leaves and stems) and belowground (roots) parts and weighed. Subsamples of leaves, stems, and roots were immediately immersed in liquid N_2_ and stored at −80°C for plant NSC (i.e., sucrose, soluble sugar, and starch), root SM (i.e., glycyrrhizin, liquiritin, total flavonoids, and total saponins) measurements and root RNA extraction. A 0.5-g root subsample was retained to estimate mycorrhizal colonization. The remaining leaves, stems, and roots were then dried at 60°C for 72 h to record dry weight (DW) and used for elemental (C, N, and P) analysis. Given the small plant size at 60 DAI, the available plant material was insufficient for measurements of NSCs and SMs. Thus, two of the four replicates were merged to create a composite sample, reducing the replicate number to three per treatment.

Mycorrhizal colonization was assessed using the method of Phillips and Hayman (1970) on roots stained with trypan blue (for more details, refer to Xie et al., 2019). The intensity of mycorrhizal colonization (M%) and arbuscule abundance (A%) was calculated with the root fragment frequency method using the MYCOCALC software (Trouvelot et al., 1986).

### Tissue C, N, and P Concentrations

Oven-dried plant tissues were ground into powder with a ball mill (RETSCH MM400; Haan, Germany). Leaf and root P concentrations were measured using an ICP-OES system (Prodigy; Teledyne Leeman, United States) after digesting with HNO_3_ in a microwave accelerated reaction system (Mars, CEM Corp., United States). Tissue C and N concentrations were analyzed with an elemental analyzer (Vario MAX; Elementar, Germany) after milling.

### P Acquisition Efficiency and P Utilization Efficiency

Considering the potential interferences of AM structure in roots, PAE and PUE were calculated on the basis of leaf organ. PAE was calculated as the ratio of leaf P content under P deficiency (LP) and sufficient P supply (HP). PUE was defined as the amount of biomass produced per unit of acquired P (López-Arredondo et al., 2014) and calculated as the ratio of the total plant biomass to leaf P concentration.

### Non-structural Carbohydrates and C-Based Secondary Metabolite Concentrations

Tissue samples for NSC (i.e., sucrose, soluble sugar, and starch) and C-based SM (i.e., glycyrrhizin, liquiritin, total flavonoids, and total saponins) assays were obtained from a freeze-dried material that was ground into fine powder using a ball mill (RETSCH MM400; Haan, Germany). For plant shoot and root NSCs, the materials (50 mg) were transferred into microcentrifuge tubes, added with 1 ml of 80% (v/v) ethanol, and incubated at 80°C for 20 min. The extracts were then clarified by centrifugation (14,000 rpm, 10 min). Ethanol was added to the centrifugation steps twice. The supernatant was collected and pooled for soluble sugar (i.e., sucrose and total soluble sugar) analysis. The residual pellets were further extracted with 2 ml of 80% (v/v) ethanol, collected, and dried after centrifugation for starch analysis. Sucrose concentrations were determined using a K-SUFRG assay kit (Megazyme, Wicklow, Ireland) according to the manufacturer’s protocols. Soluble sugar and starch concentrations were determined with the anthrone–sulfuric acid method (Gao, 2006).

For analysis of glycyrrhizin, liquiritin, total flavonoids, and total saponins, an aliquot (100 mg) of freeze-dried and powdered root samples was extracted with 67% methanol in an ultrasonic bath (250 W, 40 kHz) at room temperature. The extract solution was cooled and filtered through a 0.45-μm filter before storage at −20°C. The glycyrrhizin and liquiritin in the root extract solution were separated and detected by high-performance liquid chromatography (Agilent-1200, United States) through an Agilent ZORBAX-Eclipse XDB-C18 column (250 mm × 4.6 mm, 5 μm) and a DAD detector (Xie et al., 2018). Total flavonoids were measured in accordance with Feng et al. (2007). In brief, methanolic extracts (250 μl) were mixed with 1 ml of 67% methanol and 0.5 ml of 10% KOH and left at room temperature for 5 min. Thereafter, optical density (OD) was determined at 334 nm. Total saponins in the methanolic extracts were assessed following the method of Lan and Wang (2007). Methanolic extracts (200 μl) were dried with nitrogen flush. The pellets were dissolved with 0.25 ml of 5% vanillin–glacial acetic acid and 0.8 ml of sulfuric acid, heated at 55°C for 20 min, and cooled to room temperature. The OD at 594 nm was then recorded. Total flavonoids and saponins were expressed as liquiritin and glycyrrhizin, respectively, equivalents per gram of the plant material on a DW basis.

### Carbon Allocation and Partitioning

Plant NSC (i.e., sucrose, soluble sugar, and starch) and SM (i.e., liquiritin, glycyrrhizin, total flavonoids, and total saponins) contents were calculated by multiplying NSC and SM concentrations by DW (data shown in Supplementary Tables 1, 2). The C content in specific NSCs and SMs was calculated by multiplying specific NSC and SM contents by their mass proportion (i.e., 0.4 for NSCs and 0.6 for SMs) (Huang et al., 2017). Non-structural C allocation to specific organs (i.e., shoots, stems, and roots) was defined as the percentage of NSC contained in a specific organ to that in the whole plant (i.e., sum of shoots, stems, and roots). Similarly, root C partitioning to specific NSC and SM was calculated as a percentage of a specific NSC and SMC content compared with the total root C content.

### Plant and Fungal Gene Expression

Two sugar transfer genes, *GlySUT2* and *GlySUT4*, were analyzed to evaluate plant C allocation and partitioning in the plants. Three *R. irregularis* symbiosis marker genes, namely, *RiTEF* (translation elongation factor, the expression of which represents the relative abundance of *R. irregularis* in roots), *RiMST2* (a monosaccharide transporter gene, the expression of which indicates plant sugar translocation to AM fungi), and *RiPT* (a phosphorus transporter gene involved in mycorrhizal P translocation to plant roots), were also measured to evaluate mycorrhizal functionality (Campos-Soriano et al., 2010; Helber et al., 2011; Hu et al., 2015).

Total root RNA was extracted using CTAB (2% CTAB, 2% PVP-40, 0.1 mol L^–1^ Tris-HCl, 0.25 mol L^–1^ EDTA, and 2 mol L^–1^ NaCl), followed by purification with MicroElute RNA Clean-Up Kit (Omega BioTek, United States) and DNase I (Takara Biotechnology Co., Ltd., Dalian, China) treatment. A NanoDrop 2000 spectrophotometer (Thermo Fisher Scientific Inc., United States) and 1% agarose gel electrophoresis were used to detect RNA quantity and quality, respectively. Complementary DNA (cDNA) was synthesized from pretreated total RNA using RevertAid First Strand cDNA Synthesis Kit (Thermo Fisher Scientific Inc., United States) and following the manufacturer’s instructions. Expression changes of the target transcripts were analyzed by quantitative real-time PCR (qRT-PCR) using the Roche LightCycler 480 II Real-Time PCR System (Roche, Switzerland), and the SYBR Green method (Power SYBR Green PCR Master Mix; Applied Biosystems Inc., United States) was used to quantify the amplification results. The thermal cycling conditions were as follows: an initial denaturation phase at 95°C for 10 s, followed by 40 cycles at 95°C for 15 s, 56°C for 60 s, and 72°C for 30 s. A melting curve was produced to monitor the specificity of amplification, and the procedure was as follows: 95°C for 10 s, 60°C for 60 s, 95°C for 15 s, and 60°C for 15 s. The primers used for gene amplification are shown in Supplementary Table 3. qRT-PCR was performed with three independent biological replicates and two technical replicates. A relative quantification of gene expression levels was performed using the comparative 2^–ΔΔCt^ method (Pfaffl, 2001). Purified RNA and RNA-free water were used as negative controls to exclude genomic DNA contamination and primer dimer production. Expression values were normalized using the housekeeping gene β-actin for the plants and *RiTEF* for the AM fungus (Helber et al., 2011; Xu et al., 2016).

### Statistical Analysis

All data were checked for normality and homogeneity of variance by Shapiro–Wilk test and Levene’s test, respectively, before statistical analysis. Root colonization intensity (M and A%) was arcsine (square root [X]) transformed. Other data were Box-Cox-transformed to fulfill the requirement for ANOVA normality and homogeneity of variance when necessary (Box and Cox, 1964). Data analyses were conducted separately for the two growth stages to eliminate the potential interferences of different plant size due to treatment effects. A two-way ANOVA was performed to examine the effects of P treatment, mycorrhizal treatment, and their interactions with plant biomass and root:shoot ratio, tissue C, N, and P concentrations and N:P ratio, proportion of NSC allocation belowground, root C partitioning among root components (i.e., NSC-sucrose, soluble sugar, and starch; SMs-liquiritin, glycyrrhizin, total flavonoids, and total saponins; root growth), and *GlySUT2* and *GlySUT4* expression levels. Differences among treatments were analyzed by Turkey’s HSD test. The effects of mycorrhizal treatment or plant growth stage on root colonization intensity (M and A%) and AM fungus relative abundance (*RiTEF* expression), PAE, and AM symbiosis- related genes expression (*RiMST2* and *RiPT*) were analyzed by Student’s *t*-test. Pearson correlation analyses were performed to analyze the relationship between leaf P concentration and plant total biomass, root C partitioning between NSCs and SMs, sucrose allocation to roots, *GlySU2* and *GlySUT4* expression, and AM symbiosis-related traits (i.e., M and A%, *RiTEF*, *RiMST2*, and *RiPT* expressions). Partial correlation analyses were conducted to check the relationship of root C partitioning between root growth and NSCs or SMs. An analysis of covariance (ANCOVA) was performed to test the difference in the linear regressions between leaf P concentration and plant biomass. The results of all the analyses were considered statistically significant at a 0.05 probability level. All the statistical analyses were carried out using the software IBM SPSS Statistics version 19.0 (IBM Corp., Armonk, NY, United States).

## Results

### Arbuscular Mycorrhizal Colonization and Plant Biomass

**FIGURE 1:**
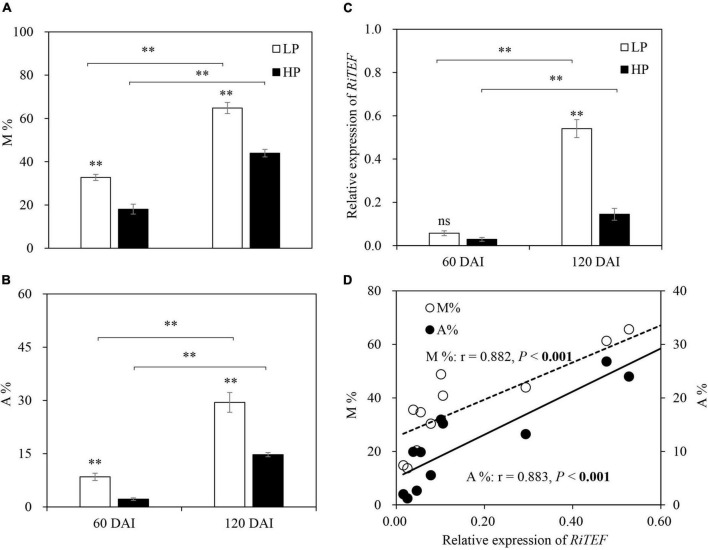
**(A)** Mycorrhizal colonization (M%), **(B)** arbuscule abundance (A%), and **(C)** relative expression of *RiTEF* as affected by soil P levels 60 and 120 days after inoculation (DAI). LP and HP represent 30 and 170 mg kg^–1^ P application, respectively. Data are presented as mean ± standard error (SE, *n* = 4). Treatment effects were tested by Student’s *t*-test at the *P* < 0.05 level. **P < 0.01. **(D)** Relative expression of *RiTEF* in relation to M and A% across two harvests. Pearson correlation analyses were conducted to check the correlations.

No mycorrhizal colonization was observed in −M roots. For +M plants, root colonization intensity (M%) and arbuscule abundance (A%) under LP were significantly higher than those under HP. As expected, M and A% were significantly increased with plant growth (Figures 1A,B). The expression of *RiTEF* generally showed a similar trend with M and A% (Figure 1C), and significant positive correlations were found between *RiTEF* expression and M or A% (Figure 1D).

**FIGURE 2:**
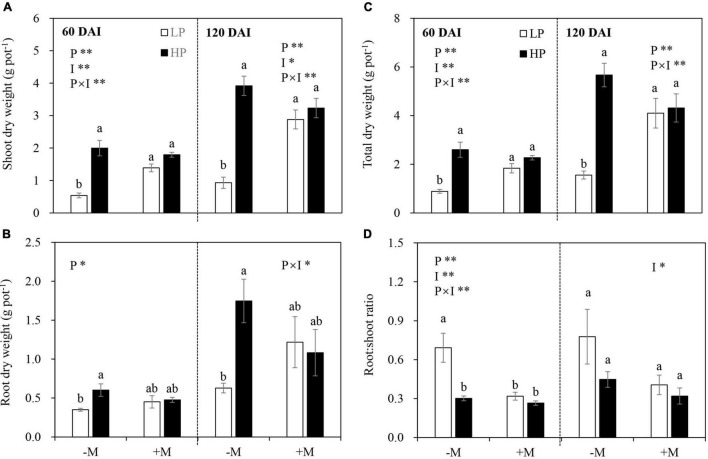
**(A)** Shoot dry weight, **(B)** root dry weight, **(C)** total dry weight, and **(D)** root:shoot ratio as affected by soil P levels (P) and mycorrhizal status (I) 60 and 120 days after inoculation (DAI). –M and +M represent non-AM and AM inoculation with *Rhizophagus irregularis*, respectively. LP and HP represent 30 and 170 mg kg^–1^ P application, respectively. Data are presented as mean ± standard error (SE, *n* = 4). Treatment effects were tested by two-way ANOVA separately between the two harvests. *P < 0.05; **P < 0.01. Multi-comparison was performed across all treatments at the same harvest time, and the same letter indicates no significant difference between the means at *P* < 0.05 by Turkey’s HSD test.

In general, P limitation significantly reduced plant shoot, root, and total DW, and this effect was dependent on mycorrhizal status in both growth stages (Figures 2A–C and Supplementary Table 4). Specifically, reductions in shoot, root, and total biomass by LP in the −M plants became significant and more pronounced than those in the +M plants. The shoot and total DW of the +M plants were significantly higher than those of the −M plants under LP, whereas no significant difference was observed under HP regardless of plant growth stage (Figures 2A–C and Supplementary Table 4).

### Plant N and P Status, P Acquisition Efficiency and P Utilization Efficiency

**FIGURE 3:**
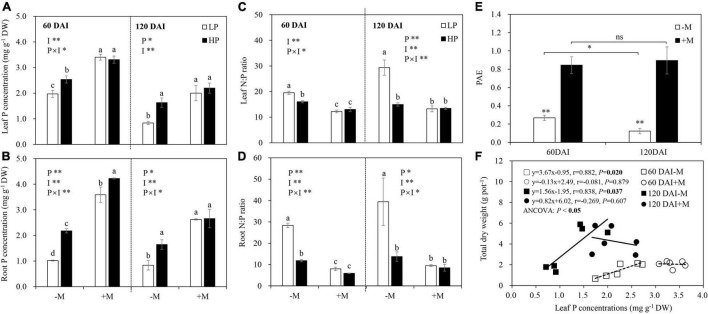
**(A)** Leaf P concentration, **(B)** root P concentration, **(C)** leaf N:P ratio, **(D)** root N:P ratio, and **(E)** phosphate acquisition efficiency (PAE) as affected by soil P levels (P) and mycorrhizal status (I) 60 and 120 days after inoculation (DAI). DW represents dry weight. –M and +M represent non-AM and AM inoculation with *R. irregularis*, respectively. LP and HP represent 30 and 170 mg kg^–1^ P application, respectively. Data are presented as mean ± standard error (SE, *n* = 3). Treatment effects were tested by two-way ANOVA separately between the two harvests. *P < 0.05; **P < 0.01. Multi-comparison was performed across all treatments at the same harvest time, and the same letter indicates no significant difference between the means at *P* < 0.05 by Turkey’s HSD test. **(F)** Leaf P concentration in relation to total dry weight for –M and +M plants at two harvests separately. Pearson correlation analyses were conducted to check the correlations.

P limitation generally significantly decreased leaf and root P concentrations, with these decreases being significant in the −M plants regardless of plant growth stage. Besides, a significant decrease in root P by LP was observed in the +M plants. AM inoculation significantly increased leaf and root P, with these increases being significant under both LP and HP in the early growth stage but only under LP in the later growth stage (Figures 3A,B and Supplementary Table 4). Leaf and root N concentrations were not affected by P level or mycorrhizal treatments in both plant growth stages (data not shown). Leaf and root N:P ratios were generally increased by LP regardless of plant growth stage (except for leaf N:P ratio at 60 DAI), but significant increases were found only in the −M plants. AM inoculation significantly reduced leaf and root N:P ratios, and these decreases were significant under both LP and HP in the early growth stage but only significant under LP in the later growth stage (Figures 3C,D and Supplementary Table 4).

The non-mycorrhizal plants showed significant decreases in PAE as they grew, while no significant difference was found in the +M plants between the two plant growth stages. AM inoculation significantly increased plant PAE regardless of plant growth stage (Figure 3E). PUE was the slope of the linear relationship between leaf P concentration and plant biomass. Significant positive correlations between leaf P concentration and total biomass were found in the −M plants in both growth stages (60 DAI: *r* = 0.882, *P* = 0.02; 120 DAI: *r* = 0.838, *P* = 0.037), and the PUE of the −M plants was increased with plant growth, as indicated by the greater slope in the late growth stage (120 DAI) than in the early growth stage (60 DAI; ANCOVA, *P* < 0.05; Figure 3F).

### C Allocation to Roots

In the early growth stage (60 DAI), the −M plants allocated more biomass to roots (shown by a higher R:S ratio) under LP versus HP, but this was not observed in the +M plants. The mycorrhizal plants generally allocated less biomass to roots (i.e., lower R:S ratio) than the −M plants, but this effect was significant only under LP (Figure 2D). In the late growth stage (120 DAI), AM inoculation decreased biomass allocation to roots. P addition and the interaction between AM inoculation and P addition showed no effects (Figure 2D and Supplementary Table 4).

**FIGURE 4:**
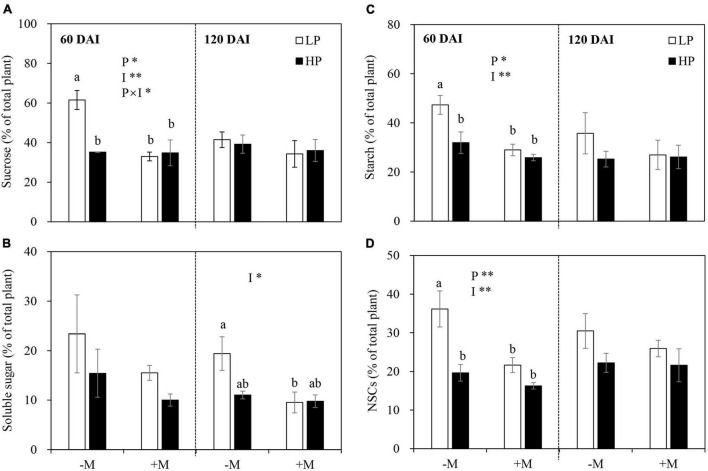
**(A)** Percentage proportion of sucrose, **(B)** soluble sugar, **(C)** starch, and **(D)** total non-structural NSCs [allocation to roots as affected by soil P levels (P) and mycorrhizal status (I)] 60 and 120 days after inoculation (DAI). –M and +M represent non-AM and AM inoculation with *R. irregularis*, respectively. LP and HP represent 30 and 170 mg kg^–1^ P application, respectively. Data are presented as mean ± standard error (SE, *n* = 3). Treatment effects were tested by two-way ANOVA separately between the two harvests. Only significant effects by ANOVA are displayed. *P < 0.05; **P < 0.01. Multi-comparison was performed across all treatments at the same harvest time, and the same letter indicates no significant difference between the means at *P* < 0.05 by Turkey’s HSD test.

In the early growth stage, the allocation of sucrose, starch, and total NSCs but not soluble sugar to roots were significantly increased by LP, and these were more evident in the −M plants versus +M plants. On the contrary, AM inoculation significantly decreased sucrose, starch, and total NSC allocation to roots (Figure 4 and Supplementary Table 4). In the late growth stage, no significant effect was observed on the allocation of all C forms to roots regardless of P treatment and mycorrhizal status, except for a decrease in soluble sugar allocation to roots by AM symbiosis (Figure 4 and Supplementary Table 4).

### Root C Partitioning Patterns and Trade-Offs Among Root Components

**FIGURE 5:**
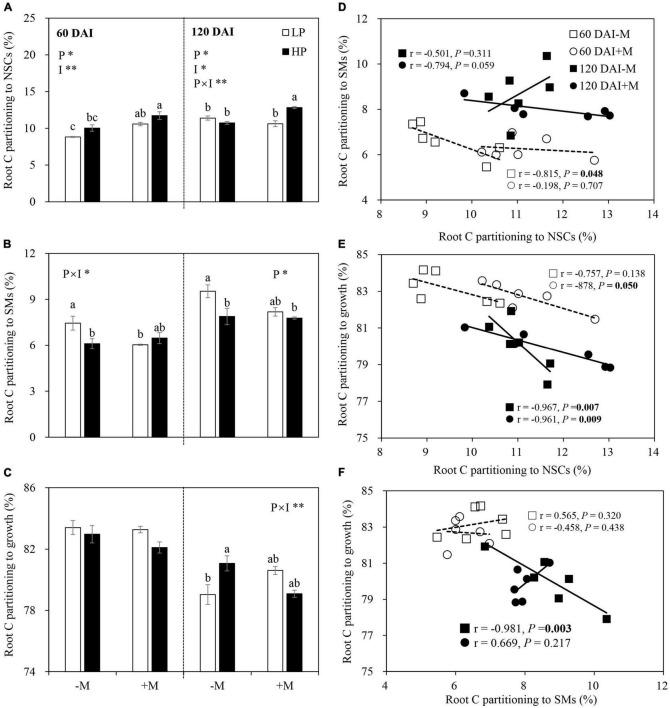
Percentage proportion of root C partitioning to **(A)** NSCs, **(B)** secondary metabolites (SMs), and **(C)** root growth as affected by soil P levels (P) and mycorrhizal status (I) 60 and 120 days after inoculation (DAI). –M and +M represent non-AM and AM inoculation with *R. irregularis*, respectively. LP and HP represent 30 and 170 mg kg^–1^ P application, respectively. Data are presented as mean ± standard error (SE, *n* = 3). Treatment effects were tested by two-way ANOVA separately between the two harvests. Only significant effects by ANOVA are displayed. *P < 0.05; **P < 0.01. Multi-comparison was performed across all treatments at the same harvest time, and the same letter indicates no significant difference between the means at *P* < 0.05 by Turkey’s HSD test. Root C partitioning to NSCs in relation to C partitioning to SMs **(D)**, root growth **(E)**, and root C partitioning to growth in relation to C partitioning to SMs **(F)** for –M and +M plants at two harvests separately. Pearson correlation analyses or partial correlation analyses were conducted to check the correlations.

The proportion of root C partitioning to total NSCs (soluble sugar + starch) was significantly lower under LP versus HP but higher in the +M plants than in the −M plants in the early growth stage (60 DAI). P treatment and mycorrhizal treatment effects on root C partitioning to NSCs in the late growth stage (120 DAI) were similar with those in the early growth stage, while the decrease in C partitioning to NSCs by LP was only observed in the +M plants, and an increase in C partitioning to NSCs by AM symbiosis was found under HP (Figure 5A and Supplementary Table 4). Root C partitioning to SMs (total flavonoids + total saponins) showed different patterns compared with NSCs. P limitation (LP) generally increased root C partitioning to SMs, and the increase became more significant in the −M plants than in the +M plants regardless of plant growth stage. A decrease in root C partitioning to SMs by AM inoculation was observed only under LP in the early growth stage (Figure 5B and Supplementary Table 4). In the early growth stage, the proportion of root C partitioning to growth was not affected by P treatment or mycorrhizal status. By the late growth stage, a significant decrease in C partitioning to root growth was observed under LP versus HP in −M plants but not in +M plants (Figure 5C and Supplementary Table 4).

A significant negative correlation was observed between root -NSCs and SMs (*r* = −0.815, *P* = 0.048) (Figure 5D). Significant negative correlations between NSCs and root growth were also observed in the +M plants in the early growth stage (*r* = −0.878, *P* = 0.05) and in the −M and +M plants (−M plants: *r* = −0.967, *P* = 0.007; +M plants: *r* = −0.961, *P* = 0.009) in the late growth stage (Figure 5E). Root C partitioning between growth and SMs showed a significant negative correlation (*r* = −0.981, *P* = 0.003) in the −M plants in the late growth stage (Figure 5F).

### Gene Expressions Related to C Allocation and Partitioning Between Host Plant and Mycorrhizal Symbiont

**FIGURE 6:**
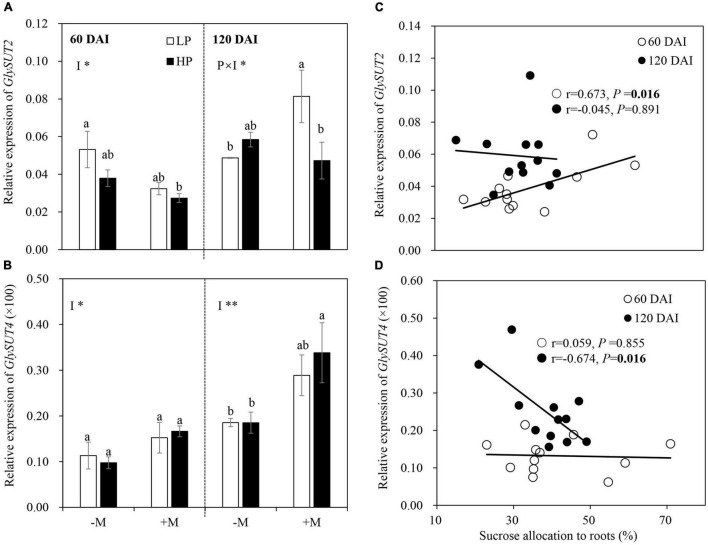
Relative expression of **(A)** *GlySUT2* and **(B)** *GlySUT4* as affected by soil P levels (P) and mycorrhizal status (I) 60 and 120 days after inoculation (DAI). –M and +M represent non-AM and AM inoculation with *R. irregularis*, respectively. LP and HP represent 30 and 170 mg kg^–1^ P application, respectively. Data are presented as mean ± standard error (SE, *n* = 3). Treatment effects were tested by two-way ANOVA separately between the two harvests. *P < 0.05; **P < 0.01. Multi-comparison was performed across all treatments at the same harvest time, and the same letter indicates no significant difference between the means at *P* < 0.05 by Turkey’s HSD test. Sucrose allocation to roots in relation to the relative expression of genes **(C)** *GlySUT2* and **(D)** *GlySUT4* at two harvests separately. Pearson correlation analyses were conducted to check the correlations.

The expression of *GlySUT2* was not affected by LP in the early growth stage regardless of mycorrhizal status, but a significant positive correlation (*r* = 0.673, *P* = 0.016) was found between *GlySUT2* expression and sucrose allocation belowground (Figures 6A,C). In the late growth stage, the expression of *GlySUT2* was significantly upregulated by LP in the +M plants, but no significant difference was observed in the −M plants. No significant correlation was found between *GlySUT2* expression and sucrose allocation to roots in this stage (Figures 6A,C and Supplementary Table 4). LP showed no significant effect on the expression of *GlySUT4* regardless of plant growth stage, but a significant negative correlation (*r* = −0.674, *P* = 0.016) was found between *GlySUT4* expression and sucrose allocation belowground in the late growth stage (Figures 6B,D and Supplementary Table 4). AM inoculation downregulated *GlySUT2* expression in the early growth stage but upregulated *GlySUT4* expression in both plant growth stages (Figures 6A,B).

**FIGURE 7:**
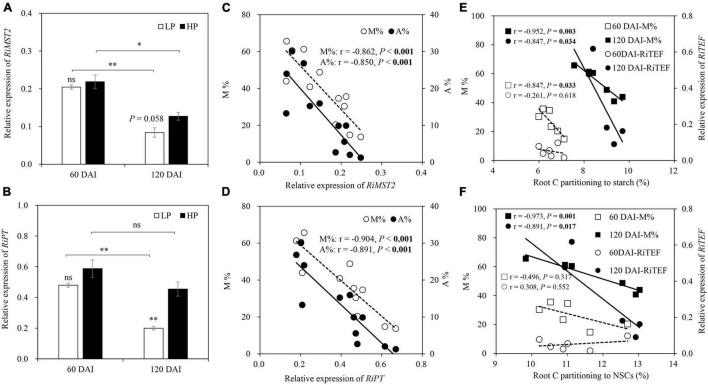
Relative expression of **(A)** *RiMST2* and **(B)** *RiPT* as affected by soil P levels 60 and 120 days after inoculation (DAI). LP and HP represent 30 and 170 mg kg^–1^ P application, respectively. Data are presented as mean ± standard error (SE, *n* = 3). Treatment effects were tested by Student’s *t*-test at the *P* < 0.05 level. *P < 0.05; **P < 0.01; ns, not significant. M and A% in relation to the relative expression of **(C)** *RiMST2* and **(D)** *RiPT* for +M plants across two harvests. M% and *RiTEF* expression in relation to root C partitioning to **(E)** starch and **(F)** NSCs for the –M and +M plants at two harvests separately. Pearson correlation analyses were conducted to check the correlations.

No expression of *RiMST2* and *RiPT* was observed in −M roots. The expression of *RiMST2* and *RiPT* in the +M plants showed a similar trend to P status, with significant or marginally significant (*P* = 0.058) decreases by LP in the late growth stage but no significant effects in the early growth stage. *RiMST2* and *RiPT* expressions were generally downregulated as the plants grew, except no significant difference in *RiPT* expression under HP between the two growth stages (Figures 7A,B). Significant negative correlations were found between *RiMST2* or *RiPT* expression and mycorrhizal colonization (M and A%; Figures 7C,D). Significant negative correlations were also found between root starch (NSCs) and M% (*r* = −0.847, *P* = 0.033) in the early growth stage and between root NSCs and M% (starch: *r* = −0.952, *P* = 0.003; NSCs: *r* = −0.973, *P* = 0.001) or *RiTEF* expression (starch: *r* = −0.847, *P* = 0.034; NSCs: *r* = −0.891, *P* = 0.017) in the late growth stage (Figures 7E,F).

## Discussion

**FIGURE 8:**
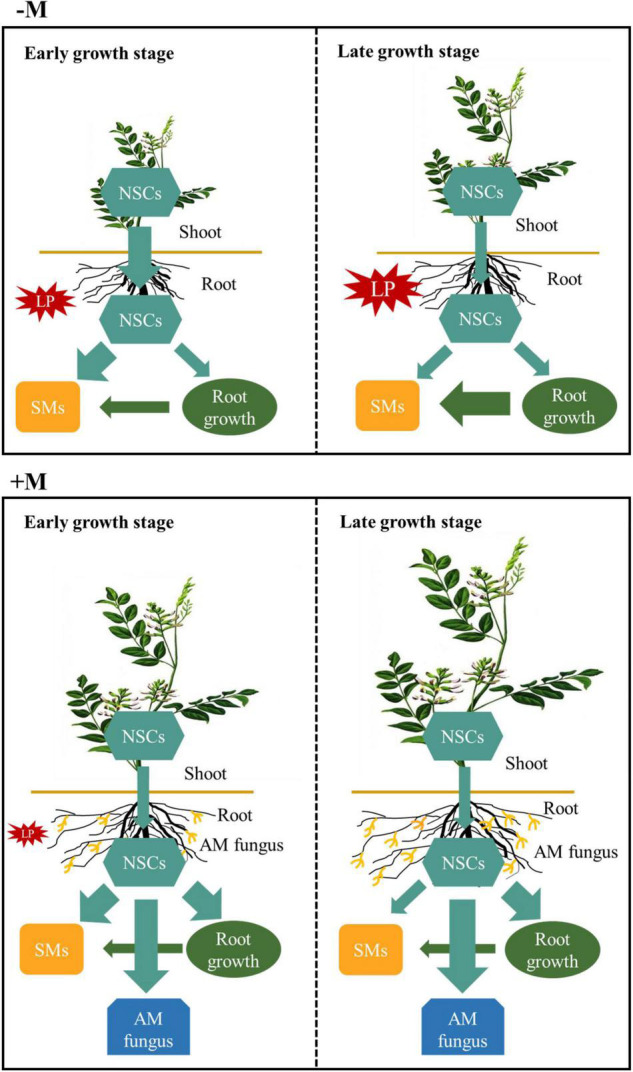
A schematic summary of carbon (C) allocation and partitioning patterns in *Glycyrrhiza uralensis* plants. For non-mycorrhizal (–M) plants, transport of non-structural carbohydrates (NSCs; light blue arrows and boxes) from shoots to roots was increased (broad arrows), along with more root NSC partitioning to secondary metabolites (SMs; yellow boxes) under phosphorus (P) limitation (red explosion) in the early growth stage; in the late growth stage, the –M plants suffered more severe P limitation (enhanced red explosion), which resulted in more root growth C (dark green arrows and boxes) partitioning to SMs without any change shoot NSC allocation to roots. For the mycorrhizal (+M) plants, transport of NSCs from aboveground to belowground did not change under P limitation for both plant growth stages, but more root C was partitioned to SMs, root growth, and AM fungus (dark blue boxes) at the expense of NSCs in the early growth stage. However, when P deficiency stress was relieved in the late growth stage, the +M plants partitioned more NSCs to root growth and AM fungus instead of SMs.

P deficiency could severely affect plant growth, development, and productivity (Hernández and Munné-Bosch, 2015). In order to improve P nutrition, plants have to integrate physiological modifications, such as modulating C allocation and partitioning in plants, and recruit root symbiotic microbes such as AM fungi to enhance external P uptake from soil and internal P-use (López-Arredondo et al., 2014). AM symbiosis could substantially improve plant P nutrition by increasing P uptake (Smith and Read, 2008), but this process largely depends on plant growth stage and mycorrhizal symbiosis development (Hart and Reader, 2002; Bao et al., 2019; van’t Padje et al., 2021). Our previous study demonstrated that well-established AM symbiosis could substantially improve licorice growth and facilitate glycyrrhizin and liquiritin accumulation under P limitation (Xie et al., 2018), mainly attributed to improved plant P nutrition by AM symbiosis (Xie et al., 2019). In this study, we investigated mycorrhizal effects on plant growth under P deficiency at two growth stages, representing the less developed AM symbiosis but high plant P demand stage (Li et al., 2006; Watts-Williams et al., 2022) and AM symbiosis well-established stage, respectively. We hypothesized that plants would suffer P deficiency even with AM symbiosis especially in the early growth stage, thus resulting in increase in internal P recycling by modulating C allocation and partitioning. As expected, the experimental results showed that plant growth stage and AM symbiosis did affect plant C allocation belowground and partitioning among root NSCs, SMs, and growth, thus affecting plant tolerance to P deficiency (Figure 8).

### Mycorrhizal Symbiosis Relieved Plant P Deficiency Regardless of Plant Growth Stage

Using critical tissue P concentration to evaluate plant nutrition status largely depends on plant growth stage; therefore, leaf N:P ratio is used instead. In general, an N:P ratio below 14 indicates N limitation, whereas an N:P ratio above 16 implies P limitation (Koerselman and Meuleman, 1996; Köhl et al., 2016). In this study, −M plants under LP had lower tissue P concentrations, higher N:P ratios (N:P ratio > 16), and lower biomass than plants grown under HP regardless of plant growth stage (Figures 2A–C, 3A–D), suggesting that the plants grown under LP were P-limited. Furthermore, tissue N:P ratio and the difference in biomass between LP and HP plants were increased as the plants grew (Figures 2, 3C,D), indicating that the −M plants experienced intensified P deficiency with plant growth. Moreover, the limiting threshold of leaf P concentration in the –M plants (approximately 2.5 mg g^–1^ in early growth stage and 1.5 mg g^–1^ in late growth stage; Figure 3F) confirmed that the plants had higher P demand in the early growth stage than in the late growth stage, which has been well-proved in cultivated licorice and in wheat and maize plants (Schilling et al., 1998; Zhou and Jin, 2016; Ven et al., 2019). We also confirmed that mycorrhizal symbiosis gradually developed with plant growth, with lower fungal root colonization (M and A%) in the early growth stage and well-established symbiosis in the late growth stage (Figures 1A,B). The experimental conditions made it possible to test our hypotheses by comparing the response of the −M and the +M plants to P deficiency in different plant growth stages.

Interestingly, the decreased tissue P concentration, increased N:P ratio, and reduced biomass under LP versus HP in the −M plants largely disappeared in the +M plants regardless of plant growth stage (Figures 2, 3A–D), suggesting that P deficiency was relieved in the +M plants under LP. These results were not completely in agreement with earlier research studies where AM plants such as wheat (*Triticum aestivum*), maize (*Zea mays*), and clover (*Medicago truncatula*) still showed lower tissue P concentration and slower plant growth under P limitation versus P sufficiency (Li et al., 2006; Sawers et al., 2017; Nguyen et al., 2019). The results also denied the hypothesis that the +M plants in the early growth stage could suffer from P deficiency because of less developed mycorrhizal symbiosis (Hart and Reader, 2002; Miller et al., 2014). As reported, positive mycorrhizal effects on plant biomass and tissue P concentration could be observed only when roots were extensively colonized by AM fungi (Schweiger et al., 2014; Tomczak and Müller, 2017). The reason for the different observations may be because licorice belongs to Leguminosae, which has high dependency on AM symbiosis (Xie et al., 2019), and minor fungal root colonization could effectively promote plant growth. Moreover, although M and A% were relatively low in the early growth stage versus the late growth stage (Figures 1A,B), AM symbiosis-functional genes *RiMST2* and *RiPT* were highly expressed and no significant difference was found between the LP and the HP plants (Figures 7A,B), suggesting the substantial C-P trade-off between host plants and AM fungi even under low mycorrhizal colonization conditions. However, it was notable that a significantly lower root P concentration in the +M plants was observed under LP compared with HP at 60 DAI (Figure 3B), confirming that the +M plants were still potentially P limited under LP versus HP in this stage.

### Carbon Allocation Belowground Depended on Plant Growth Stage and Mycorrhizal Status

It has been well-established that plants allocate more C belowground to support root growth and mycorrhizal symbiosis under P deficiency (Remy et al., 2012; Verlinden et al., 2018), whereas how plant growth stage and mycorrhizal symbiosis development affect plant C allocation is still unclear. In this study, plant C allocation to roots was increased by LP, and this increase became more significant in the −M plants in the early growth stage. However, the increased C allocation belowground disappeared in the late growth stage regardless of mycorrhizal status (Figure 4). These results were further confirmed by changes in the root:shoot ratio and expression of *GlySUT2* (Figures 2D, 6A,C), which is responsible for the transport of soluble sugar (sucrose) from shoot to root *via* phloem (Meyer et al., 2004; Boldt et al., 2011). These findings were in line with Müller et al. (2015), which showed that P-deficient white lupin (*Lupinus albus*) increased carbohydrate allocation belowground to support their growing root system as an early response to P-deficiency, while a longer period of P-deficiency leads to scavenging of P from P-containing metabolites rather than regulating C allocation belowground. The increase in C allocation belowground in the early growth stage became non-significant in the late growth stage, which may be largely attributed to the reduced P availability in the soil (Supplementary Table 5). Previous studies showed that plant C allocation belowground strongly depended on nutrient availability in soil (Poorter et al., 2012; Ven et al., 2019) and C cost for P acquisition from the soil (Janssens et al., 2010). In this study, the significant decrease in PAE observed in the late growth stage versus the early growth stage in the −M plants (Figure 3E) further confirmed the reduction of P availability in the soil. Therefore, other strategies, such as regulating C partitioning among NSCs and SMs to enhance internal P-use efficiency instead of increasing C allocation belowground, were adopted by plants to cope with intensified P deficiency (López-Arredondo et al., 2014; Müller et al., 2015). Indeed, the higher PUE observed in elder −M plants versus young plants in this study (Figure 3F) further supported this explanation. However, such changes in PUE as well as PAE with plant growth were not found in +M plants (Figures 3E,F), may be because leaf P concentrations in +M plants were above P limiting threshold, due to high PAE induced by AM symbiosis under P limitation (Figures 3A,B; Ven et al., 2019). Taken together, the results here indicated that increased C allocation belowground to cope with P deficiency was modulated by plant growth stage and AM symbiosis.

### Mycorrhizal Symbiosis Altered C Partitioning Among Different Root C Pools

In this study, P deficiency significantly increased root C partitioning to SMs, especially flavonoids, instead of NSCs, or growth of the −M plants regardless of plant growth stage (Figure 5 and Supplementary Table 6), which was consistent with the expectation and well-supported by previous studies (Keski-Saari and Julkunen-Tiito, 2003; Sampedro et al., 2011; Pant et al., 2015; Liu et al., 2016). Byrne et al. (2011) found that metabolic alterations, such as t replacement of phospholipids with sulfolipid and induction of glycolytic bypasses, were initiated after only 24 h of P deprivation. The decreases in phosphorylated metabolites, such as glucose-6-phosphate, fructose-6-phosphate, and glycerol-3-phosphate, were accompanied by increases in SMs such as flavonoids in P-deficient plant roots regardless of the duration of P deficiency (Müller et al., 2015). In contrast to the −M plants, C partitioning to total SMs in mycorrhizal root did not differ between P treatments regardless of plant growth stage (Figure 5B). Similar results were also found in pine trees colonized by ectomycorrhizal fungus *Laccaria bicolor* under P limitation, where root SMs, such as phenols and tannins, showed no significant difference between LP and HP, whereas an evident increase in these SMs in non-mycorrhizal roots was observed under P limitation (Shinde et al., 2018). These results did not support the hypothesis that more root C would be partitioned to SMs under P limitation in the early growth stage. In this study, higher PAE was observed in the +M plants than in the −M plants under P limitation (Figure 3E), indicating markedly enhanced P acquisition by AM symbiosis. The similar tissue P concentrations and biomass of the +M plants under LP versus HP also demonstrated improved P status by AM symbiosis (Figures 2, 3). Thus, it may be not economical for +M plants to invest C to SMs but instead to invest the C toward plant growth or storage in the form of NSCs to ensure long-term survival (Bloom et al., 1985; Johnson, 2010; Huang et al., 2017).

It should be noted that although root C partitioning to total SMs in the +M plants did not differ between LP and HP in both stages, C partitioning to flavonoids, including liquiritin, was generally increased by P limitation in the early growth stage (Supplementary Table 6). Phenolic compounds, such as flavonoids, are mainly biosynthesized *via* the shikimate-phenylpropanoid pathway, which is a process releasing internal P from phosphorylated metabolites, thus increasing internal PUE under P limitation (Pourcel et al., 2007; López-Arredondo et al., 2014). Moreover, flavonoids not only have been demonstrated to play key roles in scavenging free radicals produced under nutrient stress but are also directly involved in mobilization of soil phosphorus (Tomasi et al., 2008; Müller et al., 2015). Therefore, plants partitioned more root C to flavonoids when suffering from nutrient stress. However, because AM colonization intensity plays important roles affecting plant secondary metabolism, particularly in medicinal plants (Mollavali et al., 2018), differences in root C partitioning to flavonoids under LP versus under HP in the early growth stage may be simply attributable to differences in plant root AM colonization intensity (Figures 1A,B). Future studies should further investigate the underlying mechanisms of AM symbiosis in plant secondary metabolism especially under nutrient stress.

### Trade-Offs Among Root C Pools Depended on Mycorrhizal Status and Plant Growth Stage

Although trade-offs at the organ level (i.e., between above- and belowground sinks) as well as within organs (i.e., between primary and secondary metabolites) under environmental stresses, such as drought, CO_2_ elevation, and nutrient stress, have been extensively investigated in recent years (Bot et al., 2009; Huang et al., 2017, 2019; Wang et al., 2019; Zhang et al., 2022), considerably little is known about how plant growth stage and mycorrhizal symbiosis affect the trade-off patterns among C pools under nutrient stress. In this study, the increased C partitioning to SMs was at the expense of NSCs in the early growth stage but was at the cost of root growth in the late growth stage in P-deficient −M plants, as indicated by the negative correlations among these root components (Figures 5, 8). Similar results of the trade-off pattern were also observed under N deficiency conditions, where the increase of SM (total phenols and total flavonoids) accumulation in *Leymus chinensis* roots by N limitation was accompanied by decrease of NSCs (soluble sugars and starch) in early growth stage but was accompanied by decrease of root growth in the late growth stage (Wang et al., 2019). The different C partitioning patterns observed in this study between the two growth stages could be largely attributed to the increased P deficiency as the plants grew. Although the plants had lower nutrient demand in the late growth stage than in the early stage (Figure 3F), the decreased P availability in soil (Supplementary Table 5) induced more severe damage to plant growth. NSCs, such as starch and soluble sugars, not only serve as C storage pools to prepare for future challenges and active C pools to turn into SMs and growth but also act as stress protectants by scavenging of hydroxyl radicals and osmotic adjustment under nutrient stress (Pant et al., 2015; Shinde et al., 2018; Huang et al., 2019). Therefore, plants had to partition more root C to SMs even at the expense of growth to cope with intensified P limitation. Indeed, previous studies have demonstrated that resource limitation may not only force plants into trade-offs in C partitioning between structural growth, storage (NSC), and defense (SM), and that the degree of resource limitation determines the trade-off patterns (Piper et al., 2015; Zhang et al., 2022).

As expected, although the plants suffered from intensified P limitation over time, root C partitioning in the +M plants showed similar patterns between the two growth stages but evidently very different compared to that in the −M plants (Figure 8), with more C partitioning to root growth at the expense of NSCs, as indicated by the negative correlations between NSCs and root growth (Figure 5). These results were in line with an earlier report that mycorrhizal roots under LP had higher colonization intensity but lower s accumulation than those under HP, suggesting that more root NSCs were partitioned to support root and AM symbiosis development (Graham et al., 1997). This finding again demonstrated the critical roles of AM symbiosis in maintaining plant growth under nutrient stress, from the point of view of C partitioning. The increased C partitioning to root growth at the cost of NSCs in the +M plants under P limitation could be explained by the reduced C cost for P uptake and, thus, the consistently high PAE due to AM symbiosis (Ven et al., 2019).

The C-P trade-off between host plants and AM fungi influences not only plant P acquisition under P limitation but also the overall functionality of symbiosis (Smith and Read, 2008). In this study, the host plants partitioned more root C to AM symbiont at the expense of NSCs under LP versus HP in both growth stages, as indicated by the negative correlations between NSCs (including starch) and M% or *RiTEF* expression (Figures 7E,F). These results were in agreement with previous studies (Konvalinková et al., 2017; Slavíková et al., 2017), and can be explained by the fact that the C derived from soluble sugars and starch of host plants was the main C source to support AM fungus growth (Smith and Read, 2008; Gaude et al., 2012; Jiang et al., 2017). Although we did not assess AM fungal biomass, root intraradical colonization (i.e., M%) together with *RiTEF* expression could reflect plant C allocation to AM fungi, and thus serves as an indicator for evaluating fungal growth and C gain from their hosts (Charters et al., 2020; van’t Padje et al., 2021). Interestingly, although the increase in root C partitioning to the fungal symbiont was accompanied by increase in root colonization in both plant growth stages, the expression of AM symbiosis-related functional genes *RiMST2* and *RiPT* was negatively correlated with mycorrhizal colonization intensity (Figures 7C,D). The reason may be because the functional activity of AM symbiosis may not change synchronously with mycorrhizal colonization. For example, Hu et al. (2015) found the expression levels of selected symbiosis-specific genes, including *RiMST2* and *RiPT*, were not correlated with increase in AM fungal colonization. It has been demonstrated that although the intensity of AM colonization could be used to evaluate AM fungal biomass, it is often a poor predictor of mycorrhizal function (Nagy et al., 2009; Sawers et al., 2017). In addition, the negative correlations between mycorrhizal colonization and AM functional gene expression may suggest the self-regulation of mycorrhizal symbiont, as a recent study showed that plant P starvation response-centered networks regulated mycorrhizal colonization and symbiosis development (Shi et al., 2021), but further research is needed to test this hypothesis.

## Conclusion

Phosphorus limitation increased plant C allocation belowground and root C partitioning to SMs, while increased C partitioning to SMs was at the expense of NSCs in the early growth stage but at the cost of root growth in the late growth stage. However, these changes were less or not evident in the mycorrhizal plants because of the substantially improved P nutrition by AM symbiosis under P limitation. In contrast, the mycorrhizal plants partitioned more root C to support root and AM fungus growth without any changes in C allocation belowground under P limitation. The different responses in terms of C allocation and partitioning to P limitation observed between non-mycorrhizal and mycorrhizal plants may reflect the distinct strategies of plants to cope with P deficiency in different growth stages. This study also elucidated the important roles of AM fungus in helping plants adapt to nutrient stress from a new perspective, i.e., AM symbiosis mediates plant C allocation and partitioning, not only P uptake, to enhance plant tolerance to P limitation. Future studies should consider the effect of plant-AM fungi interactions with plant responses to nutrient stresses particularly under field conditions.

## Data Availability Statement

The raw data supporting the conclusion of this article will be made available by the authors, without undue reservation.

## Author Contributions

WX, ZH, and BC designed the study. WX and WF conducted the experiment and laboratory work. WX, ZH, BC, LG, and XZ contributed to data analysis. WX wrote the first draft of the manuscript. WX, AH, BC, and ZH contributed to the interpretation of the results and revised the manuscript. All authors contributed to later versions and agreed with the final version.

## Conflict of Interest

The authors declare that the research was conducted in the absence of any commercial or financial relationships that could be construed as a potential conflict of interest.

## Publisher’s Note

All claims expressed in this article are solely those of the authors and do not necessarily represent those of their affiliated organizations, or those of the publisher, the editors and the reviewers. Any product that may be evaluated in this article, or claim that may be made by its manufacturer, is not guaranteed or endorsed by the publisher.

## References

[B1] Adolfsson L. Nziengui H. Abreu I. N. Šimura J. Beebo A. Herdean A. (2017). Enhanced secondary-and hormone metabolism in leaves of arbuscular mycorrhizal *Medicago truncatula*. *Plant Physiol.* 175 392–411. 10.1104/pp.16.01509 28698354 PMC5580739

[B2] Albornoz F. E. Dixon K. W. Lambers H. (2021). Revisiting mycorrhizal dogmas: are mycorrhizas really functioning as they are widely believed to do? *Soil Ecol. Lett.* 3 73–82. 10.1007/s42832-020-0070-2

[B3] Andrino A. Guggenberger G. Sauheitl L. Burkart S. Boy J. (2021). Carbon investment into mobilization of mineral and organic phosphorus by arbuscular mycorrhiza. *Biol. Fertil. Soils* 57 47–64. 10.1007/s00374-020-01505-5

[B4] Bao X. Z. Wang Y. T. Olsson P. A. (2019). Arbuscular mycorrhiza under water-carbon-phosphorus exchange between rice and arbuscular mycorrhizal fungi under different flooding regimes. *Soil Biol. Biochem.* 129 169–177. 10.1016/j.soilbio.2018.11.020

[B5] Bloom A. J. Chapin F. S. Mooney H. A. (1985). Resource limitation in plants-an economic analogy. *Annu. Rev. Ecol. Syst.* 16 363–392. 10.1146/annurev.es.16.110185.002051

[B6] Boldt K. Pörs Y. Haupt B. Bitterlich M. Kühn C. Grimm B. (2011). Photochemical processes, carbon assimilation and RNA accumulation of sucrose transporter genes in tomato arbuscular mycorrhiza. *J. Plant Physiol.* 168 1256–1263. 10.1016/j.jplph.2011.01.026 21489650

[B7] Bot J. L. Bénard C. Robin C. Bourgaud F. Adamowicz S. (2009). The ‘trade-off’ between synthesis of primary and secondary compounds in young tomato leaves is altered by nitrate nutrition: experimental evidence and model consistency. *J. Exp. Bot.* 60 4301–4314. 10.1093/jxb/erp271 19741002

[B8] Box G. E. P. Cox D. R. (1964). An analysis of transformations. *J. R. Stat. Soc. Ser. B Methodol.* 2 211–252. 10.1111/j.2517-6161.1964.tb00553.x

[B9] Byrne S. L. Foito A. Hedley P. E. Morris J. A. Stewart D. Barth S. (2011). Early response mechanisms of perennial ryegrass (*Lolium perenne*) to phosphorus deficiency. *Ann. Bot.* 107 243–254. 10.1093/aob/mcq234 21148585 PMC3025732

[B10] Campos-Soriano L. García-Garrido J. M. Segundo B. S. (2010). Activation of basal defense mechanisms of rice plants by *Glomus intraradices* does not affect the arbuscular mycorrhizal symbiosis. *New Phytol.* 188 597–614. 10.1111/j.1469-8137.2010.03386.x 20659300

[B11] Chapin F. S. (1991). Integrated responses of plants to stress. *Bioscience* 41 29–36. 10.2307/1311538

[B12] Charters M. D. Sait S. M. Field K. J. (2020). Aphid herbivory drives asymmetry in carbon for nutrient exchange between plants and an arbuscular mycorrhizal fungus. *Curr. Biol.* 30 1801–1808. 10.1016/j.cub.2020.02.087 32275877 PMC7237887

[B13] Chen M. L. Yang G. Sheng Y. Li P. Y. Qiu H. Y. Zhou X. T. (2017). *Glomus mosseae* inoculation improves the root system architecture, photosynthetic efficiency and flavonoids accumulation of liquorice under nutrient stress. *Front. Plant Sci.* 8:931. 10.3389/fpls.2017.00931 28638391 PMC5461296

[B14] Cheng M. Li Y. Chi X. L. Li X. L. Chang D. Yang G. (2020). Analysis on international trade competitiveness of licorice extract. *Chin. Herb. Med.* 51 1970–1976. 10.7501/j.issn.0253-2670.2020.07.033

[B15] Dang H. L. Zhang T. Wang Z. K. Li G. F. Zhao W. Q. Lv X. H. (2021). Succession of endophytic fungi and arbuscular mycorrhizal fungi associated with the growth of plant and their correlation with secondary metabolites in the roots of plants. *BMC Plant Biol.* 21:165. 10.1186/s12870-021-02942-6 33820543 PMC8022407

[B16] Feng W. Wang W. Q. Zhao P. R. (2007). Study on methods in determination of general flavonoids in *Glycyrrhiza uralensis* Fisch with ultraviolet spectrophotometry. *Lishizhen Med. Mater. Med. Res.* 18 2608–2610.

[B17] Gao J. F. (2006). *Experimental Guidance for Plant Physiology.* Beijing: Higher Education Press.

[B18] Gaude N. Bortfeld S. Duensing N. Lohse M. Krajinski F. (2012). Arbuscule-containing and non-colonized cortical cells of mycorrhizal roots undergo a massive and specific reprogramming during arbuscular mycorrhizal development. *Plant J.* 69 510–528. 10.1111/j.1365-313X.2011.04810.x 21978245

[B19] Gerlach N. Schmitz J. Polatajko A. Schlüter U. Fahnenstich H. Witt S. (2015). An integrated functional approach to dissect systemic responses in maize to arbuscular mycorrhizal symbiosis. *Plant Cell Environ.* 38 1591–1612. 10.1111/pce.12508 25630535

[B20] Glynn C. Herms D. A. Oriana C. M. Hansen R. C. Larsson S. (2007). Testing the growth-differentiation balance hypothesis: dynamic responses of willows to nutrient availability. *New Phytol.* 176 623–634. 10.1111/j.1469-8137.2007.02203.x 17725548

[B21] Graham J. H. Duncan L. W. Eissenstat D. M. (1997). Carbohydrate allocation patterns in citrus genotypes as affected by phosphorus nutrition, mycorrhizal colonization and mycorrhizal dependency. *New Phytol.* 135 335–343. 10.1046/j.1469-8137.1997.00636.x

[B22] Ham B. K. Chen J. Yan Y. Lucas W. J. (2018). Insights into plant phosphate sensing and signaling. *Curr. Opin. Biotechnol.* 49 1–9. 10.1016/j.copbio.2017.07.005 28732264

[B23] Hart M. M. Reader R. J. (2002). Host plant benefit from association with arbuscular mycorrhizal fungi: variation due to differences in size of mycelium. *Biol. Fertil. Soils* 36 357–366. 10.1007/s00374-002-0539-4

[B24] Hayashi H. Sudo H. (2009). Economic importance of licorice. *Plant Biotechnol.* 26 101–104. 10.5511/plantbiotechnology.26.101

[B25] Helber N. Wippel K. Sauer N. Schaarschmidt S. Hause B. Requena N. (2011). A versatile monosaccharide transporter that operates in the arbuscular mycorrhizal fungus *Glomus* sp is crucial for the symbiotic relationship with plants. *Plant Cell* 23 3812–3823. 10.1105/tpc.111.089813 21972259 PMC3229151

[B26] Herms D. A. Mattson W. J. (1992). The dilemma of plants-to grow or defend. *Q. Rev. Biol.* 67 283–335. 10.1086/417659

[B27] Hernández I. Munné-Bosch S. (2015). Linking phosphorus availability with photo-oxidative stress in plants. *J. Exp. Bot.* 66 2889–2900. 10.1093/jxb/erv056 25740928

[B28] Hodge A. (2001). Arbuscular mycorrhizal fungi influence decomposition of, but not plant nutrient capture from, glycine patches in soil. *New Phytol.* 151 725–734. 10.1046/j.0028-646x.2001.00200.x 33853263

[B29] Hodge A. Berta G. Doussan C. Merchan F. Crespi M. (2009). Plant root growth, architecture and function. *Plant Soil* 321 153–187. 10.1007/s11104-009-9929-9

[B30] Hodge A. Helgason T. Fitter A. H. (2010). Nutritional ecology of arbuscular mycorrhizal fungi. *Fungal Ecol.* 3 267–273. 10.1016/j.funeco.2010.02.002

[B31] Hu Y. J. Wu S. L. Sun Y. Q. Li T. Zhang X. Chen C. Y. (2015). Arbuscular mycorrhizal symbiosis can mitigate the negative effects of night warming on physiological traits of *Medicago truncatula* L. *Mycorrhiza* 25 131–142. 10.1007/s00572-014-0595-2 25033924

[B32] Huang J. B. Hammerbacher A. Forkelová L. Hartmann H. (2017). Release of resource constraints allows greater carbon allocation to secondary metabolites and storage in winter wheat. *Plant Cell Environ.* 40 672–685. 10.1111/pce.12885 28010041

[B33] Huang J. B. Hammerbacher A. Weinhold A. Reichelt M. Gleixner G. Behrendt T. (2019). Eyes on the future-evidence for trade-offs between growth, storage and defense in Norway spruce. *New Phytol.* 222 144–158. 10.1111/nph.15522 30289558

[B34] Janssens I. A. Dieleman W. Luyssaert S. Subke J. A. Reichstein M. Ceulemans R. (2010). Reduction of forest soil respiration in response to nitrogen deposition. *Nat. Geosci.* 3 315–322. 10.1038/ngeo844

[B35] Jiang Y. N. Wang W. X. Xie Q. J. Liu N. Liu L. X. Wang D. P. (2017). Plants transfer lipids to sustain colonization by mutualistic mycorrhizal and parasitic fungi. *Science* 356 1172–1175. 10.1126/science.aam9970 28596307

[B36] Johnson N. C. (2010). Resource stoichiometry elucidates the structure and function of arbuscular mycorrhizas across scales. *New Phytol.* 185 631–647. 10.1111/j.1469-8137.2009.03110.x 19968797

[B37] Keski-Saari S. Julkunen-Tiito R. (2003). Early developmental responses of mountain birch (*Betula pubescens* subsp. *czerepanovii*) seedlings to different concentrations of phosphorus. *Tree Physiol.* 23 1201–1208. 10.1093/treephys/23.17.1201 14597429

[B38] Kiers E. T. Duhamel M. Beesetty Y. Mensah J. A. Franken O. Verbruggen E. (2011). Reciprocal rewards stabilize cooperation in the mycorrhizal symbiosis. *Science* 333 880–882. 10.1126/science.1208473 21836016

[B39] Kitagawa I. (2002). Licorice root. A natural sweetener and an important ingredient in Chinese medicine. *Pure Appl. Chem.* 74 1189–1198. 10.1351/pac200274071189

[B40] Kleczewski N. M. Herms D. A. Bonello P. (2010). Effects of soil type, fertilization and drought on carbon allocation to root growth and partitioning between secondary metabolism and ectomycorrhizae of *Betula papyrifera*. *Tree Physiol.* 30 807–817. 10.1093/treephys/tpq032 20462938

[B41] Koerselman W. Meuleman A. F. M. (1996). The vegetation N:P ratio: a new tool to detect the nature of nutrient limitation. *J. Appl. Ecol.* 33 1441–1450. 10.2307/2404783

[B42] Köhl L. Lukasiewicz C. E. Heijden M. G. A. (2016). Establishment and effectiveness of inoculated arbuscular mycorrhizal fungi in agricultural soils. *Plant Cell Environ.* 39 136–146. 10.1111/pce.12600 26147222

[B43] Konvalinková T. Püschel D. Řezáčová V. Gryndlerová H. Jansa J. (2017). Carbon flow from plant to arbuscular mycorrhizal fungi is reduced under phosphorus fertilization. *Plant Soil* 419 319–333. 10.1007/s11104-017-3350-6

[B44] Lan X. Wang H. X. (2007). Determination of total saponins in *Glycyrrhiza* by colorimetry. *Lishizhen Med. Mater. Med. Res.* 18 886–887.

[B45] Li H. Smith S. E. Holloway R. E. Zhu Y. Smith F. A. (2006). Arbuscular mycorrhizal fungi contribute to phosphorus uptake by wheat grown in a phosphorus-fixing soil even in the absence of positive growth responses. *New Phytol.* 172 536–543. 10.1111/j.1469-8137.2006.01846.x 17083683

[B46] Liu L. Yang D. F. Liang T. Y. Zhang H. H. He Z. G. Liang Z. S. (2016). Phosphate starvation promoted the accumulation of phenolic acids by inducing the key enzyme genes in *Salvia miltiorrhiza* hairy roots. *Plant Cell Rep.* 35 1933–1942. 10.1007/s00299-016-2007-x 27271760

[B47] López-Arredondo D. L. Leyva-González M. A. González-Morales S. I. López-Bucio J. Herrera-Estrella L. (2014). Phosphate nutrition: improving low-phosphate tolerance in crops. *Annu. Rev. Plant Biol.* 65 95–123. 10.1146/annurev-arplant-050213-035949 24579991

[B48] Malhotra H. Vandana, Sharma S. Pandey R. (2018). “Phosphorus nutrition: plant growth in response to deficiency and excess,” in *Plant Nutrients and Abiotic Stress Tolerance*, eds Hasanuzzaman M. Fujita M. Oku H. Nahar K. Hawrylak-Nowak B. (Singapore: Springer), 171–190. 10.1007/978-981-10-9044-8_7

[B49] Meyer S. Lauterbach C. Niedermeier M. Barth I. Sjolund R. D. Sauer N. (2004). Wounding enhances expression of AtSUC3, a sucrose transporter from *Arabidopsis* sieve elements and sink tissues. *Plant Physiol.* 134 684–693. 10.1104/pp.103.033399 14739351 PMC344544

[B50] Miller R. E. Gleadow R. M. Cavagnaro T. R. (2014). Age versus stage: does ontogeny modify the effect of phosphorus and arbuscular mycorrhizas on above- and below-ground defence in forage sorghum? *Plant Cell Environ.* 37 929–942. 10.1111/pce.12209 24118061

[B51] Mo X. H. Zhang M. K. Liang C. Y. Cai L. Y. Tian J. (2019). Integration of metabolome and transcriptome analyses highlights soybean roots responding to phosphorus deficiency by modulating phosphorylated metabolite processes. *Plant Physiol. Biochem.* 139 697–706. 10.1016/j.plaphy.2019.04.033 31054472

[B52] Mollavali M. Perner H. Rohn S. Riehle P. Hanschen F. S. Schwarz D. (2018). Nitrogen form and mycorrhizal inoculation amount and timing affect flavonol biosynthesis in onion (*Allium cepa* L.). *Mycorrhiza* 28 59–70. 10.1007/s00572-017-0799-3 28948352 PMC5748431

[B53] Müller J. Gödde V. Niehaus K. Zörb C. (2015). Metabolic adaptations of white lupin roots and shoots under phosphorus deficiency. *Front. Plant Sci.* 6:1014. 10.3389/fpls.2015.01014 26635840 PMC4656794

[B54] Nagy R. Drissner D. Amrhein N. Jakobsen I. Bucher M. (2009). Mycorrhizal phosphate uptake pathway in tomato is phosphorus-repressible and transcriptionally regulated. *New Phytol.* 181 950–959. 10.1111/j.1469-8137.2008.02721.x 19140941

[B55] Nasr Esfahani M. Inoue K. Nguyen K. H. Chu H. D. Watanabe Y. Kanatani A. (2021). Phosphate or nitrate imbalance induces stronger molecular responses than combined nutrient deprivation in roots and leaves of chickpea plants. *Plant Cell Environ.* 44 574–597. 10.1111/pce.13935 33145807

[B56] Nguyen T. D. Cavagnaro T. R. Watts-Williams S. J. (2019). The effects of soil phosphorus and zinc availability on plant responses to mycorrhizal fungi: a physiological and molecular assessment. *Sci. Rep.* 9:14880. 10.1038/s41598-019-51369-5 31619728 PMC6795859

[B57] Pant B. D. Pant P. Erban A. Huhman D. Kopka J. Scheible W. R. (2015). Identification of primary and secondary metabolites with phosphorus status-dependent abundance in *Arabidopsis*, and of the transcription factor PHR 1 as a major regulator of metabolic changes during phosphorus limitation. *Plant Cell Environ.* 38 172–187. 10.1111/pce.12378 24894834

[B58] Parniske M. (2008). Arbuscular mycorrhiza: the mother of plant root endosymbioses. *Nat. Rev. Microbiol.* 6 763–775. 10.1038/nrmicro1987 18794914

[B59] Parra-Londono S. Kavka M. Samans B. Snowdon R. Wieckhorst S. Uptmoor R. (2018). Sorghum root-system classification in contrasting P environments reveals three main rooting types and root-architecture-related marker-trait associations. *Ann. Bot.* 121 267–280. 10.1093/aob/mcx157 29351588 PMC5808808

[B60] Pfaffl M. W. (2001). A new mathematical model for relative quantification in real-time RT-PCR. *Nucleic Acids Res.* 29:e45. 10.1093/nar/29.9.e45 11328886 PMC55695

[B61] Phillips J. M. Hayman D. S. (1970). Improved procedures for clearing roots and staining parasitic and vesicular-arbuscular mycorrhizal fungi for rapid assessment of infection. *Trans. Br. Mycol. Soc.* 55 158–161. 10.1016/S0007-1536(70)80110-3

[B62] Piper F. I. Gundale M. J. Fajardo A. (2015). Extreme defoliation reduces tree growth but not C and N storage in a winter-deciduous species. *Ann. Bot.* 115 1093–1103. 10.1093/aob/mcv038 25851136 PMC4648455

[B63] Poorter H. Niklas K. J. Reich P. B. Oleksyn J. Poot P. Mommer L. (2012). Biomass allocation to leaves, stems and roots: meta-analyses of interspecific variation and environmental control. *New Phytol.* 193 30–50. 10.1111/j.1469-8137.2011.03952.x 22085245

[B64] Pourcel L. Routaboul J. M. Cheynier V. Lepiniec L. Debeaujon I. (2007). Flavonoid oxidation in plants: from biochemical properties to physiological functions. *Trends Plant Sci.* 12 29–36. 10.1016/j.tplants.2006.11.006 17161643

[B65] Remy E. Cabrito T. R. Batista R. A. Teixeira M. C. Sá-Correia I. Duque P. (2012). The Pht1; 9 and Pht1; 8 transporters mediate inorganic phosphate acquisition by the *Arabidopsis thaliana* root during phosphorus starvation. *New Phytol.* 195 356–371. 10.1111/j.1469-8137.2012.04167.x 22578268

[B66] Řezáčová V. Slavíková R. Zemková L. Konvalinková T. Procházková V. Št’ovíček V. (2018). Mycorrhizal symbiosis induces plant carbon reallocation differently in C_3_ and C_4_ *Panicum* grasses. *Plant Soil* 425 441–456. 10.1007/s11104-018-3606-9

[B67] Sampedro L. Moreira X. Zas R. (2011). Costs of constitutive and herbivore-induced chemical defences in pine trees emerge only under low nutrient availability. *J. Ecol.* 99 818–827. 10.1111/j.1365-2745.2011.01814.x

[B68] Sawers R. J. H. Svane S. F. Quan C. Grønlund M. Wozniak B. Gebreselassie M. N. (2017). Phosphorus acquisition efficiency in arbuscular mycorrhizal maize is correlated with the abundance of root-external hyphae and the accumulation of transcripts encoding pht1 phosphate transporters. *New Phytol.* 214 632–643. 10.1111/nph.14403 28098948

[B69] Schilling G. Gransee A. Deuhel A. Lezoviz G. Ruppel S. (1998). Phosphorus availability, root exudates, and microbial activity in the rhizosphere. *J. Plant Nutr. Soil Sci.* 161 465–478. 10.1002/jpln.1998.35816

[B70] Schweiger R. Baier M. C. Müller C. (2014). Arbuscular mycorrhiza-induced shifts in foliar metabolism and photosynthesis mirror the developmental stage of the symbiosis and are only partly driven by improved phosphate uptake. *Mol. Plant Microbe Interact.* 27 1403–1412. 10.1094/MPMI-05-14-0126-R 25162317

[B71] Schweiger R. Müller C. (2015). Leaf metabolome in arbuscular mycorrhizal symbiosis. *Curr. Opin. Plant Biol.* 26 120–126. 10.1016/j.pbi.2015.06.009 26202872

[B72] Shi J. C. Zhao B. Y. Zheng S. Zhang X. W. Wang X. L. Dong W. T. (2021). A phosphate starvation response-centered network regulates mycorrhizal symbiosis. *Cell* 184 5527–5540. 10.1016/j.cell.2021.09.030 34644527

[B73] Shinde S. Naik D. Cumming J. R. (2018). Carbon allocation and partitioning in *Populus tremuloides* are modulated by ectomycorrhizal fungi under phosphorus limitation. *Tree Physiol.* 38 52–65. 10.1093/treephys/tpx117 29036599

[B74] Slavíková R. Püschel D. Janoušková M. Hujslová M. Konvalinková T. Gryndlerová H. (2017). Monitoring CO_2_ emissions to gain a dynamic view of carbon allocation to arbuscular mycorrhizal fungi. *Mycorrhiza* 27 35–51. 10.1007/s00572-016-0731-2 27549438

[B75] Smith S. E. Jakobsen I. Grønlund M. Smith F. A. (2011). Roles of arbuscular mycorrhizas in plant phosphorus nutrition: interactions between pathways of phosphorus uptake in arbuscular mycorrhizal roots have important implications for understanding and manipulating plant phosphorus acquisition. *Plant Physiol.* 156 1050–1057. 10.1104/pp.111.174581 21467213 PMC3135927

[B76] Smith S. E. Read D. J. (2008). *Mycorrhizal Symbiosis.* London: Academic Press.

[B77] Smith S. E. Smith F. A. Jakobsen I. (2003). Mycorrhizal fungi can dominate phosphate supply to plants irrespective of growth responses. *Plant Physiol.* 133 16–20. 10.1104/pp.103.024380 12970469 PMC1540331

[B78] Tomasi N. Weisskopf L. Renella G. Landi L. Pinton R. Varanini Z. (2008). Flavonoids of white lupin roots participate in phosphorus mobilizationfrom soil. *Soil Biol. Biochem.* 40 1971–1974. 10.1016/j.soilbio.2008.02.017

[B79] Tomczak V. V. Müller C. (2017). Influence of arbuscular mycorrhizal stage and plant age on the performance of a generalist aphid. *J. Insect Physiol.* 98 258–266. 10.1016/j.jinsphys.2017.01.016 28159616

[B80] Trouvelot A. Kough J. L. Gianinazzi-Pearson V. (1986). “Mesure du taux de mycorhization VA d’un système radiculaire. Recherche de méthode d’estimation ayant une signification fonctionnelle,” in *Physiological and Genetical Aspects of Mycorrhizae: Proceedings of the 1st European Symposium on Mycorrhizae, Dijon*, eds Gianinazzi-Pearson V. Gianinazzi S. (Paris: INRA), 217–221.

[B81] van’t Padje A. Galvez L. O. Klein M. Hink M. A. Postma M. Shimizu T. (2021). Temporal tracking of quantum-dot apatite across in vitro mycorrhizal networks shows how host demand can influence fungal nutrient transfer strategies. *ISME J.* 15 435–449. 10.1038/s41396-020-00786-w 32989245 PMC8027207

[B82] Ven A. Verlinden M. S. Fransen E. Olsson P. A. Verbruggen E. Wallander H. (2020). Phosphorus addition increased carbon partitioning to autotrophic respiration but not to biomass production in an experiment with *Zea mays*. *Plant Cell Environ.* 43 2054–2065. 10.1111/pce.13785 32400909

[B83] Ven A. Verlinden M. S. Verbrugge E. Vicca S. (2019). Experimental evidence that phosphorus fertilization and arbuscular mycorrhizal symbiosis can reduce the carbon cost of phosphorus uptake. *Funct. Ecol.* 33 2215–2225. 10.1111/1365-2435.13452

[B84] Verlinden M. S. Ven A. Verbruggen E. Janssens I. A. Wallander H. Vicca S. (2018). Favorable effect of mycorrhizae on biomass production efficiency exceeds their carbon cost in a fertilization experiment. *Ecology* 99 2525–2534. 10.1002/ecy.2502 30218450

[B85] Wang B. Gong J. R. Zhang Z. H. Yang B. Liu M. Zhu C. C. (2019). Nitrogen addition alters photosynthetic carbon fixation, allocation of photoassimilates, and carbon partitioning of *Leymus chinensis* in a temperate grassland of inner mongolia. *Agric. For. Meteorol.* 279:107743. 10.1016/j.agrformet.2019.107743

[B86] Wang Y. L. Lambers H. (2020). Root-released organic anions in response to low phosphorus availability: recent progress, challenges and future perspectives. *Plant Soil* 447 135–156. 10.1007/s11104-019-03972-8

[B87] Wang Y. L. Lysoe E. Armarego-Marriott T. Erban A. Paruch L. VanEerde A. (2018). Transcriptome and metabolome analyses provide insights into root and root-released organic anion responses to phosphorus deficiency in oat. *J. Exp. Bot.* 69 3759–3771. 10.1093/jxb/ery176 29757407

[B88] Watts-Williams S. J. Gill A. R. Jewell N. Brien C. J. Berger B. Tran B. T. (2022). Enhancement of sorghum grain yield and nutrition: a role for arbuscular mycorrhizal fungi regardless of soil phosphorus availability. *Plants People Planet* 4 143–156. 10.1002/ppp3.10224

[B89] Xie W. Hao Z. P. Yu M. Wu Z. X. Zhao A. H. Li J. L. (2019). Improved phosphorus nutrition by arbuscular mycorrhizal symbiosis as a key factor facilitating glycyrrhizin and liquiritin accumulation in *Glycyrrhiza uralensis*. *Plant Soil* 439 243–257. 10.1007/s11104-018-3861-9

[B90] Xie W. Hao Z. P. Zhou X. F. Jiang X. L. Xu L. J. Wu S. L. (2018). Arbuscular mycorrhiza facilitates the accumulation of glycyrrhizin and liquiritin in *Glycyrrhiza uralensis* under drought stress. *Mycorrhiza* 28 285–300. 10.1007/s00572-018-0827-y 29455337

[B91] Xu G. J. Cai W. Gao W. Liu C. S. (2016). A novel glucuronosyltransferase has an unprecedented ability to catalyse continuous two-step glucuronosylation of glycyrrhetinic acid to yield glycyrrhizin. *New Phytol.* 212 123–135. 10.1111/nph.14039 27252088 PMC7167757

[B92] Zhang Z. H. Gong J. R. Shi J. Y. Li X. B. Song L. Y. Zhang W. Y. (2022). Multiple herbivory pressures lead to different carbon assimilation and allocation strategies: evidence from a perennial grass in a typical steppe in northern China. *Agric. Ecosyst. Environ.* 326:107776. 10.1016/j.agee.2021.107776

[B93] Zhao M. L. Zhao J. Yuan J. Hale L. Wen T. Huang Q. W. (2021). Root exudates drive soil-microbe-nutrient feedbacks in response to plant growth. *Plant Cell Environ.* 44 613–628. 10.1111/pce.13928 33103781

[B94] Zhou C. M. Jin G. Q. (2016). *Liquorice.* Beijing: China Agriculture Press.

