# Supplementary material for: Increased Carbon Partitioning to Secondary Metabolites Under Phosphorus Deficiency in Glycyrrhiza uralensis Fisch. Is Modulated by Plant Growth Stage and Arbuscular Mycorrhizal Symbiosis
Source: Front Plant Sci. 2022 Jun 2;13:876192. doi: 10.3389/fpls.2022.876192 (PMC9201690; doi:10.3389/fpls.2022.876192)
Supplement: Supplementary file 1 [file Table_1.DOCX]

**Supplementary Material**

**Increased Carbon Partitioning to Secondary Metabolites under Phosphorus Deficiency in *Glycyrrhiza Uralensis* Fisch. Is Modulated by Plant Growth Stage and Arbuscular Mycorrhizal Symbiosis**

Wei Xie^1,2^, Angela Hodge^3^, Zhipeng Hao^1, *^, Wei Fu^1,2^, Lanping Guo^4^, Xin Zhang^1^, Baodong Chen^1, 2, *^

^1^State Key Laboratory of Urban and Regional Ecology, Research Center for Eco-Environmental Sciences, Chinese Academy of Sciences, Beijing 100085, China

^2^University of Chinese Academy of Sciences, Beijing 100049, China

^3^Department of Biology, University of York, York YO10 5DD, United Kingdom

^4^National Resource Center for Chinese Materia Medica, China Academy of Chinese Medical Sciences, Beijing 100700, China

**Dataset for C Allocation and Partitioning Calculation**

The tables below (Tables S1–S2) show the leaf, stem, and root non-structural carbohydrates (NSCs) (Table S1) and root secondary metabolites (SMs) concentrations (Table S2) of *Glycyrrhiza uralensis* plants. These data were used to calculate the proportion of C allocation at the whole plant level and partitioning at the root metabolite level.

**Table S1.** Leaf, stem, and root non-structural carbohydrate (NSCs; i.e., sucrose, starch, and soluble sugar) concentrations of *Glycyrrhiza uralensis* plants at 60 and 120 days after inoculation (DAI). LP and HP represent 30 and 170 mg kg^−1^ P application, respectively. −M and +M represent non-AM and AM inoculation with *Rhizophagus irregularis*, respectively*.* DW represents dry weight. Data presented are means ± standard error (n = 3).

| Harvest time (T) | Mycorrhizal status (I) | | Phosphorus level (P) | | Sucrose (mg g^-1^ DW) | | | Starch (mg g^-1^ DW) | | | Soluble sugar (mg g^-1^ DW) | | |
| --- | --- | --- | --- | --- | --- | --- | --- | --- | --- | --- | --- | --- | --- |
|  |  |  |  |  | Leaf | Stem | Root | Leaf | Stem | Root | Leaf | Stem | Root |
| 60 DAI | -M | LP | | 4.50 ± 0.37 | | 6.16 ± 0.12 | 14.44 ± 1.64 | 59.43 ± 12.34 | 28.26 ± 6.32 | 57.44 ± 1.69 | 98.22 ± 29.76 | 104.55 ± 33.17 | 38.70 ± 1.41 |
|  |  | HP | | 6.52 ± 0.65 | | 9.10 ± 0.97 | 14.11 ± 0.82 | 53.30 ± 8.91 | 48.15 ± 5.83 | 71.44 ± 6.84 | 73.71 ± 16.35 | 90.87 ± 25.54 | 41.14 ± 1.74 |
|  | +M | LP | | 1.27 ± 0.33 | | 8.93 ± 0.73 | 9.83 ± 0.78 | 60.12 ± 1.74 | 49.43 ± 1.43 | 65.54 ± 1.89 | 68.99 ± 1.99 | 88.96 ± 2.57 | 46.14 ± 1.33 |
|  |  | HP | | 0.76 ± 0.38 | | 4.94 ± 0.78 | 8.10 ± 2.59 | 59.76 ± 2.68 | 50.13 ± 3.11 | 71.55 ± 1.66 | 100.04 ± 2.13 | 113.36 ± 1.12 | 47.46 ± 7.52 |
| 120 DAI | -M | LP | | 9.50 ± 1.69 | | 13.44 ± 1.42 | 13.59 ± 0.50 | 131.33 ± 6.93 | 87.32 ± 5.03 | 89.71 ± 3.37 | 118.44 ± 14.1 | 78.79 ± 7.13 | 34.32 ± 3.15 |
|  |  | HP | | 7.98 ± 1.94 | | 15.11 ± 0.29 | 15.19 ± 1.67 | 119.87 ± 11.69 | 82.92 ± 13.71 | 88.68 ± 1.20 | 132.64 ± 6.08 | 66.46 ± 0.87 | 33.87 ± 2.23 |
|  | +M | LP | | 9.77 ± 1.41 | | 17.10 ± 1.79 | 15.98 ± 0.87 | 99.26 ± 2.55 | 73.85 ± 4.01 | 89.89 ± 2.79 | 146.99 ± 18.56 | 75.19 ± 4.71 | 30.13 ± 2.90 |
|  |  | HP | | 7.73 ± 0.41 | | 15.76 ± 1.65 | 17.61 ± 1.20 | 96.44 ± 4.07 | 78.19 ± 2.51 | 102.17 ± 1.76 | 128.15 ± 12.84 | 76.55 ± 6.75 | 38.11 ± 1.02 |

**Table S2.** Root secondary metabolite (i.e., liquiritin, glycyrrhizin, total flavonoid, and total saponin) concentrations in *Glycyrrhiza uralensis* plants at 60 and 120 days after inoculation (DAI). LP and HP represent 30 and 170 mg kg^−1^ P application, respectively. −M and +M represent non-AM and AM inoculation with *Rhizophagus irregularis*, respectively*.* DW represents dry weight. Data presented are means ± standard error (n = 3).

| Harvest time (T) | Mycorrhizal status (I) | Phosphorus level (P) | Liquiritin (mg g^-1^ DW) | Glycyrrhizin (mg g^-1^ DW) | Total flavonoid (mg g^-1^ DW) | Total saponin (mg g^-1^ DW) |
| --- | --- | --- | --- | --- | --- | --- |
| 60 DAI | -M | LP | 0.53 ± 0.04 | 1.06 ± 0.16 | 11.54 ± 0.88 | 43.62 ± 2.75 |
|  |  | HP | 0.20 ± 0.01 | 0.68 ± 0.05 | 6.68 ± 0.48 | 38.90 ± 2.65 |
|  | +M | LP | 0.21 ± 0.01 | 0.54 ± 0.06 | 5.60 ± 0.14 | 37.60 ± 0.24 |
|  |  | HP | 0.11 ± 0.01 | 0.30 ± 0.08 | 3.95 ± 0.30 | 41.71 ± 2.76 |
| 120 DAI | -M | LP | 0.31 ± 0.11 | 1.10 ± 0.32 | 19.08 ± 0.07 | 52.46 ± 2.41 |
|  |  | HP | 1.06 ± 0.29 | 2.55 ± 0.70 | 13.73 ± 1.16 | 47.93 ± 2.58 |
|  | +M | LP | 0.49 ± 0.01 | 2.64 ± 0.05 | 15.03 ± 1.14 | 48.32 ± 0.88 |
|  |  | HP | 0.80 ± 0.26 | 1.38 ± 0.16 | 13.49 ± 0.37 | 45.43 ± 0.41 |

**RNA Extraction and Plant and Fungal Gene Expression Analysis**

**Table S3.** PCR primers used in this study. *RiMST2, Rhizophagus irregularis* sugar transporter gene; *RiPT, R. irregularis* phosphate transporter gene; *RiTEF*, *R. irregularis* elongation factor 1-alpha gene, an AM fungi reference gene; *GlySUT2, Glycyrrhiza uralensis* sucrose transporter gene 2; *GlySUT4,* *G.* *uralensis* sucrose transporter gene 4; *β-actin*, plant reference gene.

| Genes | Accession No. | Forward (5’→3’) | Reverse (5’→3’) | Reference |
| --- | --- | --- | --- | --- |
| **Fungal genes** | | | | |
| *RiMST2* | XM_025331163.1 | GGCAGGATATTTGTCTGATAG | GCAATAACTCTTCCCGTATAC | Helber et al. (2011) |
| *RiPT* | XM_025315184.1 | CGCGTTGGATATTGCTTTTT | GAGGACAGCGAAACCCATTA | Campos-Soriano et al. (2010) |
| *RiTEF* | XM_025321412.1 | TGTTGCTTTCGTCCCAATATC | GGTTTATCGGTAGGTCGAG | Helber et al. (2011) |
| **Plant genes** | | | | |
| *GlySUT2* | Glyur000433s00026964.1 | TCGTCTTTGTTTGCATGGCT | CGAACACAACCAAGGAAGCA | this study |
| *GlySUT4* | Glyur000011s00000684.1 | TGGGTTTGTATGGGGAATCT | TCAATGCAGCTATCACGATG | this study |
| *β-actin* | EU190972.1 | CCAGTGCTTCTAACTGAG | CAATACCAGTTGTACGA | Xu et al. (2016) |

The primers of *GlySUT2* and *GlySUT4* used in this study were designed by Primer 3.0 (Whitehead Institute). In brief, the conserved sequences of 7-8 homologous species of these genes were first identified in accordance with the *Glycyrrhiza uralensis* genome database (<http://ngs-data-archive.psc.riken.jp/Gur-genome/search-result.pl>). Primer 3.0 was used to design primers, and NCBI Primer Blast was used for specificity verification. PCR products were checked by agarose gel electrophoresis, taking the generation of only one single band of the expected size as a criterion for specificity, followed by Sanger sequencing to ensure that the right target gene fragment was amplified.

**Data Analysis**

**Table S4.** ANOVA results for the data shown in figures. DW, dry weight; **P**, phosphorus application level; **I**, mycorrhizal status. *, *P* < 0.05; **, *P* < 0.01; ns, not significant.

| **Item** | **Early growth stage (60 DAI)** | | | **Late growth stage (120 DAI)** | | |
| --- | --- | --- | --- | --- | --- | --- |
|  | **P** | **I** | **P × I** | **P** | **I** | **P × I** |
| **Plant growth related** | | | | | | |
| Shoot dry weight (g pot^-1^) | ***F*_1,16_=51.96^**^** | ***F*_1,16_=16.02^**^** | ***F*_1,16_=23.03^**^** | ***F*_1,16_=37.89^**^** | ***F*_1,16_=5.42^*^** | ***F*_1,16_=23.64^**^** |
| Root dry weight (g pot^-1^) | ***F*_1,16_=7.59^*^** | *F*_1,16_=0.00^ns^ | *F*_1,16_=3.84^ns^ | *F*_1,16_=3.48^ns^ | *F*_1,16_=0.02^ns^ | ***F*_1,16_=5.67^*^** |
| Total dry weight (g pot^-1^) | ***F*_1,16_=45.78^**^** | ***F*_1,16_=10.19^**^** | ***F*_1,16_=19.46^**^** | ***F*_1,16_=19.36^**^** | *F*_1,16_=1.46^ns^ | ***F*_1,16_=15.70^**^** |
| Root:shoot ratio | ***F*_1,16_=24.85^**^** | ***F*_1,16_=20.25^**^** | ***F*_1,16_=10.10^**^** | *F*_1,16_=2.98^ns^ | ***F*_1,16_=5.60^*^** | *F*_1,16_=0.41^ns^ |
| Leaf P concentration (mg g^-1^ DW) | *F*_1,12_=3.15^ns^ | ***F*_1,12_=69.41^**^** | ***F*_1,12_=6.24^*^** | ***F*_1,12_=5.85^*^** | ***F*_1,12_=18.04^**^** | *F*_1,12_=2.16^ns^ |
| Root P concentration (mg g^-1^ DW) | ***F*_1,12_=98.44^**^** | ***F*_1,12_=418.17^**^** | ***F*_1,12_=39.80^**^** | ***F*_1,12_=5.18^*^** | ***F*_1,12_=32.50^**^** | ***F*_1,12_=5.20^*^** |
| Leaf N:P ratio | *F*_1,12_=4.37**^ns^** | ***F*_1,12_=65.13^**^** | ***F*_1,12_=11.24^*^** | ***F*_1,12_=18.53^**^** | ***F*_1,12_=28.45^**^** | ***F*_1,12_=19.58^**^** |
| Root N:P ratio | ***F*_1,12_=169.35^**^** | ***F*_1,12_=338.04^**^** | ***F*_1,12_=102.21^**^** | ***F*_1,12_=9.38^*^** | ***F*_1,12_=23.88^**^** | ***F*_1,12_=5.22^*^** |
| **C allocation and partitioning pattern related** |  |  |  |  |  |  |
| Sucrose allocation belowground (%) | ***F*_1,12_=8.44^*^** | ***F*_1,12_=11.77^**^** | ***F*_1,12_=11.13^*^** | *F*_1,12_=0.00^ns^ | *F*_1,12_=0.96^ns^ | *F*_1,12_=0.13^ns^ |
| Soluble sugar allocation belowground (%) | *F*_1,12_=3.20^ns^ | *F*_1,12_=2.01^ns^ | *F*_1,12_=0.01^ns^ | *F*_1,12_=3.64^ns^ | ***F*_1,12_=6.80^*^** | *F*_1,12_=4.12^ns^ |
| Starch allocation belowground (%) | ***F*_1,12_=8.34^*^** | ***F*_1,12_=14.46^**^** | *F*_1,12_=3.63^ns^ | *F*_1,12_=0.93^ns^ | *F*_1,12_=0.45^ns^ | *F*_1,12_=0.67^ns^ |
| Total NSCs allocation belowground (%) | ***F*_1,12_=20.72^**^** | ***F*_1,12_=12.20^**^** | *F*_1,12_=2.77^ns^ | *F*_1,12_=3.21^ns^ | *F*_1,12_=0.54^ns^ | *F*_1,12_=0.31^ns^ |
| Root C partitioning to NSCs (%) | ***F*_1,12_=10.73^*^** | ***F*_1,12_=22.94^**^** | *F*_1,12_=0.01^ns^ | ***F*_1,12_=7.95^*^** | ***F*_1,12_=5.78^*^** | ***F*_1,12_=26.74^**^** |
| Root C partitioning to SMs (%) | *F*_1,12_=1.80^ns^ | *F*_1,12_=2.42^ns^ | ***F*_1,12_=7.01^*^** | ***F*_1,12_=7.76^*^** | *F*_1,12_=3.93^ns^ | *F*_1,12_=2.86^ns^ |
| Root C partitioning to growth (%) | *F*_1,12_=3.56^ns^ | *F*_1,12_=1.39^ns^ | *F*_1,12_=0.75^ns^ | *F*_1,12_=0.34^ns^ | *F*_1,12_=0.22^ns^ | ***F*_1,12_=16.26^**^** |
| *GlySUT2* | *F*_1,12_=4.01^ns^ | ***F*_1,12_=10.76^*^** | *F*_1,12_=0.41^ns^ | *F*_1,12_=2.08^ns^ | *F*_1,12_=0.83^ns^ | ***F*_1,12_=7.83^*^** |
| *GlySUT4* | *F*_1,12_=0.00^ns^ | ***F*_1,12_=5.06^*^** | *F*_1,12_=0.38^ns^ | *F*_1,12_=0.24^ns^ | ***F*_1,12_=14.11^**^** | *F*_1,12_=0.35^ns^ |

**Table S5** Soil available P concentration as affected by mycorrhizal status (I) and harvest time (T) under LP and HP conditions. −M and +M represent non-AM and AM inoculation with *Rhizophagus irregularis*, respectively. LP and HP represent 30 and 170 mg kg^−1^ P application, respectively. 60 DAI and 120 DAI represent 60 and 120 days after inoculation (DAI), respectively. Treatment effects were tested by two-way ANOVA. Means ± standard errors (n = 4) followed by the same letter within a column are not statistically different at *P* < 0.05 by Turkey’s HSD test. *, *P* < 0.05; **, *P* < 0.01.

| Phosphorus level | Mycorrhizal status (I) | Harvest time (T) | Soil available P (mg kg^-1^) |
| --- | --- | --- | --- |
| LP | -M | 60 DAI | 9.67 ± 0.23 a |
|  |  | 120 DAI | 8.13 ± 0.35 b |
|  | +M | 60 DAI | 7.60 ± 0.22 b |
|  |  | 120 DAI | 2.78 ± 0.50 c |
| ANOVA |  | I | ***F*_1,16_=116.24^**^** |
|  |  | T | ***F*_1,16_=85.55^**^** |
|  |  | I × T | ***F*_1,16_=22.73^**^** |
| HP | -M | 60 DAI | 48.83 ± 0.87 a |
|  |  | 120 DAI | 36.19 ± 1.00 c |
|  | +M | 60 DAI | 41.23 ± 0.85 b |
|  |  | 120 DAI | 33.17 ± 1.08 c |
| ANOVA |  | I | ***F*_1,16_=30.92^**^** |
|  |  | T | ***F*_1,16_=117.07^**^** |
|  |  | I × T | ***F*_1,16_=5.73^*^** |

**Table S6** Proportion (%) of root C partitioning among NSCs (i.e., sucrose, soluble sugar, and starch) and SMs (i.e., liquiritin, glycyrrhizin, total flavonoids, and total saponins) as affected by soil P levels (P) and mycorrhizal status (I) at 60 and 120 days after inoculation (DAI). −M and +M represent non-AM and AM inoculation with *Rhizophagus irregularis*, respectively. LP and HP represent 30 and 170 mg kg^−1^ P application, respectively. Treatment effects were tested by two-way ANOVA. Means ± standard errors (n = 3) followed by the same letter within a column are not statistically different at *P* < 0.05 by Turkey’s HSD test. *, *P* < 0.05; **, *P* < 0.01; ns, not significant.

| Harvest time | Mycorrhizal status (I) | Phosphorus level (P) | NSCs (%) | | | SMs (%) | | | |
| --- | --- | --- | --- | --- | --- | --- | --- | --- | --- |
|  |  |  | Sucrose | Soluble sugar | Starch | Liquiritin | Glycyrrhizin | Total flavonoids | Total saponins |
| 60 DAI | -M | LP | 1.34 ± 0.16 a | 3.59 ± 0.14 b | 5.24 ± 0.08 b | 0.074 ± 0.11 a | 0.148 ± 0.02 a | 1.53 ± 0.12 a | 5.92 ± 0.34 |
|  |  | HP | 1.33 ± 0.08 a | 3.87 ± 0.16 ab | 6.17 ± 0.32 a | 0.028 ± 0.00 bc | 0.096 ± 0.01 ab | 0.91 ± 0.07 b | 5.39 ± 0.37 |
|  | +M | LP | 0.93 ± 0.08 a | 4.37 ± 0.10 ab | 6.21 ± 0.14 a | 0.030 ± 0.00 b | 0.076 ± 0.01 b | 0.77 ± 0.02 bc | 5.27 ± 0.04 |
|  |  | HP | 0.77 ± 0.25 a | 4.91 ± 0.18 a | 6.83 ± 0.16 a | 0.015 ± 0.00 d | 0.043 ± 0.01 b | 0.55 ± 0.04 c | 5.93 ± 0.40 |
| ANOVA |  | P | *F*_1,12_=0.31^ns^ | *F*_1,12_=2.65^ns^ | ***F*_1,12_=15.24^**^** | ***F*_1,12_=94.68^**^** | ***F*_1,12_=10.38^*^** | ***F*_1,12_=32.92^**^** | *F*_1,12_=0.04^ns^ |
|  |  | I | ***F*_1,12_=9.49^*^** | ***F*_1,12_=14.70^**^** | ***F*_1,12_=17.04^**^** | ***F*_1,12_=83.79^**^** | ***F*_1,12_=22.10^**^** | ***F*_1,12_=60.41^**^** | *F*_1,12_=0.03^ns^ |
|  |  | P × I | *F*_1,12_=0.22^ns^ | *F*_1,12_=0.09^ns^ | *F*_1,12_=0.62^ns^ | ***F*_1,12_=25.91^**^** | *F*_1,12_=0.48^ns^ | ***F*_1,12_=7.57^*^** | *F*_1,12_=3.36^ns^ |
| 120 DAI | -M | LP | 1.23 ± 0.05 | 3.26 ± 0.20 ab | 8.13 ± 0.29 b | 0.033 ± 0.01 c | 0.150 ± 0.04 a | 2.55 ± 0.03 a | 6.98 ± 0.39 |
|  |  | HP | 1.38 ± 0.15 | 2.84 ± 0.10 bc | 7.90 ± 0.11 b | 0.170 ± 0.01 a | 0.347 ± 0.09 a | 1.72 ± 0.18 b | 6.17 ± 0.37 |
|  | +M | LP | 1.45 ± 0.08 | 2.58 ± 0.15 c | 8.05 ± 0.25 b | 0.066 ± 0.00 bc | 0.359 ± 0.01 a | 1.98 ± 0.15 b | 6.21 ± 0.12 |
|  |  | HP | 1.61 ± 0.11 | 3.48 ± 0.08 a | 9.35 ± 0.20 a | 0.110 ± 0.04 ab | 0.190 ± 0.02 a | 1.74 ± 0.07 b | 6.04 ± 0.06 |
| ANOVA |  | P | *F*_1,12_=2.21^ns^ | *F*_1,12_=2.99^ns^ | ***F*_1,24_=5.65^*^** | ***F*_1,12_=27.32^**^** | *F*_1,12_=0.16^ns^ | ***F*_1,12_=18.63^**^** | *F*_1,12_=3.14^ns^ |
|  |  | I | *F*_1,12_=4.77^ns^ | *F*_1,12_=0.02^ns^ | ***F*_1,24_=9.27^*^** | *F*_1,12_=0.29^ns^ | *F*_1,12_=0.85^ns^ | ***F*_1,12_=5.10^*^** | *F*_1,12_=2.64^ns^ |
|  |  | P × I | *F*_1,12_=0.01^ns^ | ***F*_1,12_=22.49^**^** | ***F*_1,24_=11.42^*^** | ***F*_1,12_=10.35^*^** | ***F*_1,12_=8.66^*^** | ***F*_1,12_=5.74^*^** | *F*_1,12_=1.38^ns^ |
